# Prehospital immune responses and development of multiple organ dysfunction syndrome following traumatic injury: A prospective cohort study

**DOI:** 10.1371/journal.pmed.1002338

**Published:** 2017-07-18

**Authors:** Jon Hazeldine, David N. Naumann, Emma Toman, David Davies, Jonathan R. B. Bishop, Zhangjie Su, Peter Hampson, Robert J. Dinsdale, Nicholas Crombie, Niharika Arora Duggal, Paul Harrison, Antonio Belli, Janet M. Lord

**Affiliations:** 1 Institute of Inflammation and Ageing, University of Birmingham, Birmingham, United Kingdom; 2 NIHR Surgical Reconstruction and Microbiology Research Centre, Queen Elizabeth Hospital Birmingham, Birmingham, United Kingdom; 3 Scar Free Foundation, Birmingham Centre for Burns Research, Birmingham, United Kingdom; 4 Midlands Air Ambulance, Unit 16 Enterprise Trading Estate, Brierley Hill, West Midlands, United Kingdom; Oregon Health and Science University, UNITED STATES

## Abstract

**Background:**

Almost all studies that have investigated the immune response to trauma have analysed blood samples acquired post-hospital admission. Thus, we know little of the immune status of patients in the immediate postinjury phase and how this might influence patient outcomes. The objective of this study was therefore to comprehensively assess the ultra-early, within 1-hour, immune response to trauma and perform an exploratory analysis of its relationship with the development of multiple organ dysfunction syndrome (MODS).

**Methods and findings:**

The immune and inflammatory response to trauma was analysed in 89 adult trauma patients (mean age 41 years, range 18–90 years, 75 males) with a mean injury severity score (ISS) of 24 (range 9–66), from whom blood samples were acquired within 1 hour of injury (mean time to sample 42 minutes, range 17–60 minutes). Within minutes of trauma, a comprehensive leukocytosis, elevated serum pro- and anti-inflammatory cytokines, and evidence of innate cell activation that included neutrophil extracellular trap generation and elevated surface expression of toll-like receptor 2 and CD11b on monocytes and neutrophils, respectively, were observed. Features consistent with immune compromise were also detected, notably elevated numbers of immune suppressive CD16^BRIGHT^ CD62L^DIM^ neutrophils (82.07 x 10^6^/l ± 18.94 control versus 1,092 x 10^6^/l ± 165 trauma, *p* < 0.0005) and CD14^+^HLA-DR^low/−^ monocytes (34.96 x 10^6^/l ± 4.48 control versus 95.72 x 10^6^/l ± 8.0 trauma, *p* < 0.05) and reduced leukocyte cytokine secretion in response to lipopolysaccharide stimulation. Exploratory analysis via binary logistic regression found a potential association between absolute natural killer T (NKT) cell numbers and the subsequent development of MODS. Study limitations include the relatively small sample size and the absence of data relating to adaptive immune cell function.

**Conclusions:**

Our study highlighted the dynamic and complex nature of the immune response to trauma, with immune alterations consistent with both activation and suppression evident within 1 hour of injury. The relationship of these changes, especially in NKT cell numbers, to patient outcomes such as MODS warrants further investigation.

## Abstract 2

In this prospective cohort study, Jon Hazeldine and colleagues investigate the immune and inflammatory response of trauma patients immediately after and in the hours and days following injury.

## Author summary

**Why was this study done?:**

- Whilst it is recognised that traumatic injury elicits a profound immune and inflammatory response, our knowledge is based almost entirely upon the analysis of blood samples acquired from patients post-hospital admission.
- Very little is known with regards to the immune and inflammatory status of trauma patients in the immediate aftermath of injury, thereby limiting our ability to determine factors influencing patient outcome, stratification for treatment, and the development of novel therapeutics.
- This study was undertaken to provide information on the ultra-early immune and inflammatory response that occurs within minutes of traumatic injury.

**What did the researchers do and find?:**

- We analysed the composition and function of immune cells and the concentrations of cytokines in peripheral blood samples acquired from 89 adult trauma patients within 1 hour of injury as well as 4–12 and 48–72 hours postinjury.
- We found traumatic injury resulted in immediate immune dysfunction, with evidence of concomitant immune activation and suppression detected within minutes of injury.
- Our work uncovered a dynamic nature to the very early post-trauma immune response, revealing that certain features detected in blood samples acquired within minutes of injury were absent from subsequent samples obtained in the hours and days post-trauma.

**What do these findings mean?:**

- Immune cell activation and the generation of inhibitory cells occur within minutes of injury, supporting the notion of a concomitant induction of immune activation and suppression immediately after trauma.
- The complex nature of the acute immune response to injury suggests that targeting 1 element of the immune response is unlikely to reduce immuneparesis or the incidence of multiple organ dysfunction syndrome (MODS).

## Introduction

Although the major and immediate cause of death following severe trauma is haemorrhage, many trauma victims later die following complications such as multiorgan dysfunction or sepsis, with the individual’s immune response to injury significantly influencing the chances of developing these life-threatening conditions [1]. Two opposing clinical syndromes characterise the immune and inflammatory response to traumatic injury: systemic inflammatory response syndrome (SIRS), characterised by elevated levels of circulating proinflammatory cytokines and immune cell activation, and compensatory anti-inflammatory response syndrome (CARS), characterised by raised anti-inflammatory cytokines and immuneparesis [2]. The immune response that develops during the SIRS and CARS responses is complex and involves the innate and adaptive arms of the immune system, with significant alterations apparent in the composition, phenotype, and/or function of the circulating immune cell pools. For example, following major injury, marked alterations have been described in the antimicrobial functions of neutrophils [3–6], the surface phenotype of monocytes [7,8], and the absolute number of circulating lymphocytes [9].

The current paradigm for how major injury influences the immune system is based almost entirely upon the analysis of blood samples obtained from patients post-hospital admission and several hours postinjury. Indeed, with the exception of a small number of studies in which research samples were acquired at the scene of injury [10–12], the literature is saturated with studies in which emergency departments or intensive care units (ICUs) have served as the site of initial sample collection, an approach that has resulted in significant interstudy variation in time to first blood sampling [4,6,7,13–17]. Thus, whilst we have a detailed understanding of the alterations that occur in the immune system during the acute immune response to injury, our knowledge of trauma-induced changes in immunity during the ultra-early postinjury phase (particularly within the first hour) is limited. Indeed, of the above-mentioned prehospital based studies, only 1 investigated immune function, reporting a significant impairment in lipopolysaccharide (LPS)-induced cytokine production by whole blood leukocytes within minutes of injury, suggesting that trauma patients are immune suppressed even prior to hospital admission [10]. Unfortunately, the group performed no additional assays to investigate the function of specific immune cells, nor did they examine whether traumatic injury resulted in any immediate alterations to the composition or surface phenotype of the circulating immune cell pool. Such a study would provide much-needed and novel data relating to the immune status of trauma patients prior to their arrival at hospital and would provide the evidence base for early intervention to improve patient outcomes or stratification for treatment.

Results presented in a series of recent prospective observational cohort studies suggest that patients who experience poor clinical outcomes following traumatic injury elicit a more robust and prolonged immune/inflammatory response than those who report better outcomes [18–22]. These data, coupled with studies that have shown that elevated proinflammatory cytokines [22–26], impaired leukocyte function [13,27], and altered monocyte phenotype [28,29] are associated with and/or predictive of mortality, multiple organ dysfunction/failure, and sepsis, suggest a potential role for immune monitoring in identifying patients at risk of poor outcome. Common to all these studies has been the acquisition and analysis of post-hospital admission blood samples, meaning the data collected relates to the immune and inflammatory response during the acute postinjury phase. Recently, in a cohort of 40 patients, from whom blood samples were acquired within 2 hours of injury, Manson et al. [9] reported an increased percentage of CD56^DIM^ natural killer (NK) cells and a reduced frequency of γδ–low T lymphocytes in those subjects who subsequently developed multiple organ dysfunction syndrome (MODS). Thus, it would appear that immunological events activated prior to hospital admission may impact upon patient recovery [9].

Here, via the analysis of blood samples acquired from 89 adult trauma patients within 1 hour of injury (mean time to sampling; 42±1 minutes postinjury), we have analysed the composition, phenotype, and/or function of the innate and adaptive arms of the immune system to provide a comprehensive insight into the immediate cellular immune response to trauma. Using these data, an exploratory analysis was performed to test for potential relationships between the ultra-early immune/inflammatory response to injury and the development of MODS. In addition to the samples collected within 1 hour of injury, we also analysed the immune status of patients 4–12 and 48–72 hours postinjury, intervals chosen to mimic the time periods in which previous trauma studies had acquired their first postinjury research blood samples [6,16,18–22].

## Methods

### Study design and setting

A prospective observational study was undertaken at a regional trauma network in the West Midlands, United Kingdom. The study aimed to characterise the relationship between biomarkers, brain injury severity, and outcome. Patients were enrolled into the study during prehospital emergency evacuation and were followed up at the major trauma centre to which they were conveyed, the Queen Elizabeth Hospital Birmingham. Research ethics committee approval was granted before the study was started (Brain Biomarkers After Trauma Cohort Study; reference 13/WA/0399). No patients in the study received prehospital blood products.

### Patient selection

On a 24/7 basis between 15 May 2014 and 16 December 2016, prehospital emergency care teams acquired blood samples from adult trauma patients (≥18 years) with a suspected injury severity score (ISS) ≥ 8 within 1 hour of injury (defined as the time of phone call to emergency services). A screening log of all trauma patients was prospectively recorded in order to reduce the risk of selection bias. All patients had complete follow-up data for their hospital stay.

### Capacity and consent

Because of the nature of their injuries, patients were unlikely to be able to provide informed consent to enrol in the study. Recruitment into the study was therefore undertaken under the guidance of the Mental Health Capacity Act for research in emergency situations, in accordance with the Declaration of Helsinki. If the patient lacked capacity, a written agreement for study participation was sought from a legal consultee, with written consent obtained from the patient after they regained capacity. In cases in which the patient did not regain capacity to consent, data were retained in accordance with the legal consultee’s assent.

### Prehospital enrolment and blood sampling

As this study involved the acquisition of blood samples during the prehospital evacuation of trauma patients, regional training for prehospital personnel was undertaken before the study was started. They were instructed to sample any patient with significant injuries and a suspected ISS greater than 8 that warranted immediate transfer to a major trauma centre and were provided with information on how to acquire, store, and hand over blood samples for research. Peripheral venous blood was either obtained during the intravenous cannulation of patients or by venepuncture. Blood tubes were stored in the ambulance at room temperature until arrival at hospital, when they were placed into a study-specific refrigerator. All samples were collected and the analysis was begun within 1 hour of deposition by the same laboratory researcher on a 24/7 basis in order to minimise heterogeneity in blood preparation and storage. Further samples were taken at 4–12 and 48–72 hours postinjury by research nursing staff, who delivered samples directly to the laboratory. At all time points, blood samples were collected into 3 separate BD Vacutainers (Becton Dickinson, Oxford, UK) containing lithium heparin, z-serum clotting activator, or 1/10 volume of 3.2% trisodium citrate. Patients were excluded from the study if they were deemed unlikely to survive transportation to hospital. Patients who had prehospital blood samples taken >1 hour postinjury, a confirmed ISS < 8, or a previous diagnosis of neurodegenerative disease were also subsequently excluded from the study. Data obtained from isolated traumatic brain injury (TBI) patients and subjects who received steroid treatment were not included in the final analysis.

### Healthy controls

Blood was sampled from 116 adults who served as healthy controls (HCs). The mean age and gender of the HC cohort were not significantly different from those of the patient cohort (S1 Table). HCs were volunteers who were not taking any regular medication for a diagnosed illness and did not have an acute episode of infection. Healthy subjects were excluded if they were taking any medication that would modify immune responses, such as steroids.

### Data collection

Clinical and demographic data were obtained from electronic medical records as well as a contemporaneous history provided by the next of kin. Data regarding mortality, length of ICU and hospital stay, ISS, new ISS (NISS), and abbreviated injury scale scores were obtained from the Trauma Audit Research Network (a UK-based centralised network that records injury details).

### Haematological analysis

Whole blood cell counts were performed on citrated whole blood using a Sysmex XN-1000 haematology analyser (Sysmex UK, Milton Keynes, UK), which defines immature granulocytes (IGs) as promyelocytes, myelocytes, and metamyelocytes. Instrument performance was ensured by daily measurement of quality control material (XN Check) and participation in an external quality assurance scheme (UKNEQAS, Watford, UK).

### Neutrophil oxidative burst

The ability of neutrophils to generate reactive oxygen species (ROS) in response to stimulation with 1.62 μM phorbol 12-myristate 13-acetate (PMA) was assessed using the commercially available PhagoBURST kit (BD Biosciences, Oxford, UK) in 100 μl aliquots of heparinised whole blood. Ten thousand neutrophils, gated according to forward scatter (FS)/sideward scatter (SS) properties, were analysed on an Accuri C6 flow cytometer. Data were evaluated using CFlow software (BD Biosciences) and are presented as the percentage of neutrophils that produced ROS as well as their mean fluorescence intensity (MFI).

### Neutrophil phenotyping

Fifty μl aliquots of whole blood were stained for 30 minutes on ice with the following mouse antihuman monoclonal antibodies or their concentration-matched isotype controls: 8 μg/ml fluorescein isothiocyanate (FITC)-labelled CD62L (clone DREG56; eBioscience), 4 μg/ml CXCR1-FITC (clone eBIO8F1-1-4; eBioscience), 20 μg/ml R-phycoerythrin (PE)-labelled CD88 (clone S5/1; BioLegend, London, UK), 2 μg/ml CXCR2-PE (clone eBio5E8-C7-F10; eBioscience), 20 μg/ml CD63-PE (clone CLB-180; Life Technologies, Cheshire, UK), 4 μg/ml APC-labelled CD11b (clone ICRF44, BioLegend), or 2 μg/ml CD16-APC (clone 3G8, BD Biosciences). Post-incubation, red blood cells were lysed (BD PharmLyse, BD Biosciences) and samples analysed using an Accuri C6 flow cytometer, where receptor expression on 10,000 neutrophils was recorded as median fluorescence intensity (MedFI).

To determine neutrophil responsiveness to formylated peptides, heparinised blood was stimulated for 5 minutes (37°C, 5% CO_2_) with 1 μM formyl-methionine-leucine-phenylalanine (fMLF), after which CD62L and CD11b immunostaining was performed.

### Detection of citrullinated histone H3 (CitH3) in platelet-free plasma (PFP)

PFP was prepared by double centrifugation of citrated whole blood. Blood was centrifuged at 2,000 x g for 20 minutes at 4°C, and the top two-thirds of platelet-poor plasma (PPP) was removed. PPP was then centrifuged at 13,000 x g for 2 minutes at 4°C, after which PFP was collected and stored at −80°C. CitH3 in PFP was measured by western blotting as described previously [3].

### Monocyte and lymphocyte phenotyping

Fifty or one hundred μl aliquots of heparinised whole blood were stained on ice for 30 minutes with combinations of the following mouse antihuman monoclonal antibodies or their concentration-matched isotype controls: 1 μg/ml CD14-FITC (clone TUK4; Dako, Cambridgeshire, UK), 0.5 μg/ml CD16-FITC (clone eBioCB16; eBioscience), 2.5 μg/ml CCR7-FITC (clone 150503; R&D Systems, Abingdon, Oxford, UK), 1 μl CD8-PE (clone UCHT-4; ImmunoTools, Friesoythe, Germany), 0.5 μg/ml CD14-PE (clone 61D3; eBioscience), 2 μg/ml CD19-PE (clone HIB19; eBioscience), 5 μl CD56-PE (clone AF12-7H3; Miltenyi Biotec, Surrey, UK), 0.07 μg/ml HLA-DR-PE (clone LN3; eBioscience), 0.1 μg/ml CD45-APC (clone HI100; BioLegend), 10 μg/ml CD86-APC (clone IT2.2; BioLegend), 10 μg/ml toll like receptor (TLR)4-APC (clone HTA125; eBioscience), 0.625 μg/ml TLR2-APC (clone 11G7; BD Biosciences), 4 μg/ml Pacific Blue (PcB)-labelled CD3 (clone UCHT1; BD Biosciences), 1.5 μg/ml CD4-PcB (clone OKT4; eBioscience), 4 μg/ml CD14-PcB (clone M5E2; BioLegend), or 2 μg/ml PE-Cy7-labelled CD3 (clone UCHT1; eBioscience). Postincubation, red blood cells were lysed (BD PharmLyse, BD Biosciences) and samples fixed for 20 minutes at room temperature (RT) with 50 μl of fixation medium (Life Technologies). After a single wash in phosphate-buffered saline, samples were analysed on a CyAn_ADP_ bench top cytometer (Dako) and data evaluated using Summit v4.3 software (Dako). Monocytes were defined as CD14^+^, B cells as CD19^+^, NK cells as CD3^−^56^+^, and NKT cells as CD3^+^56^−^. T cells were defined as CD3^+^ cells and divided into 4 subsets based on the differential surface expression of the protein tyrosine phosphatase isoform CD45RA and the chemokine receptor CCR7. These subsets were denoted as naive (CD45RA^+^ CCR7^+^), central memory (CD45RA^−^ CCR7^+^), effector memory (CD45RA^−^ CCR7^−^), and terminally differentiated effector memory (TEMRA; CD45RA^+^ CCR7^−^) cells. Receptor expression on a minimum of 1,000 monocytes was recorded as both the percentage of antigen-positive cells and MedFI. B cell, T cell, NK cell, and NKT cell frequencies were determined in a total of 5,000 lymphocytes. These frequency values were used alongside whole blood cell counts from the Sysmex XN-1000 haematology analyser to calculate the absolute numbers of immune cells.

### LPS stimulation of whole blood and cytokine/chemokine quantification

Four hundred μl aliquots of heparinised whole blood were stimulated with 1 or 10 ng/ml LPS from *Escherichia coli* (serotype 0111:B4; Sigma-Aldrich, Dorset, UK) or vehicle control for 4 hours (37°C, 5% CO_2_). Postincubation, samples were centrifuged at 461 x g for 8 minutes at 4°C, after which supernatants were collected and stored at −80°C until analysed. Following the manufacturer’s instructions, concentrations of tumour necrosis factor-alpha (TNF-α), interleukin (IL)-6, IL-8, IL-10, and monocyte chemoattractant protein-1 (MCP-1) were quantified using a commercially available magnetic bead 5-plex assay (BioRad, Hertfordshire, UK). Data were analysed using BioPlex software (BioRad), and cytokine/chemokine concentrations were normalised to monocyte counts.

### Cytokine and cortisol measurements

Blood collected into BD vacutainers containing z-serum clotting activator was left at RT for 30 minutes prior to centrifugation at 1,620 x g for 10 minutes at 4°C, after which serum was removed and stored at −80°C until analysed. Following the manufacturer’s instructions, concentrations of IL-1 receptor antagonist (IL-1Ra), IL-6, IL-8, IL-10, TNF-α, granulocyte-colony stimulating factor (G-CSF), and MCP-1 were measured using a commercially available magnetic bead multiplex immunoassay (BioRad), whilst cortisol concentrations were measured by an enzyme-linked immunosorbent assay (IBL international, Hamburg, Germany).

### Outcomes

The primary outcome of interest was the development of MODS, which was defined as a Sequential Organ Failure Assessment score of 6 or more, on 2 or more consecutive days, at least 48 hours postadmission [9]. Secondary outcomes were mortality and ICU-free days and hospital-free days (as calculated by 30 minus the number of days the patient stayed in hospital).

### Statistical analysis

The current study is an exploratory investigation using a small convenience sample of trauma patients in order to generate hypotheses. There was no hypothesised effect upon which to power the study. Data were checked for normality using the Shapiro-Wilk test. A one-way ANOVA with Bonferroni post hoc test or a Kruskal-Wallis test with Dunn’s post hoc test was used to assess differences between patients and HCs. Relationships between continuous variables were assessed using a Pearson’s correlation. Comparisons of MODS versus no MODS patients were made on 34 variables; differences in continuous variables were assessed by Mann-Whitney U tests or independent samples *t* tests, whilst Chi-squared tests were performed to compare categorical variables. The resulting *p*-values from these 34 tests were compared to their Benjamini-Hochberg critical values to control for a false discovery rate of 5% [30]. Binary logistic regression analyses were used to explore the relationships between immune parameters and the development of MODS. In these models, the reference level of MODS was coded as “No MODS” (versus “MODS”). Model performance was measured through the proportion of variation explained by the model via R^2^ statistics and Brier scores, the level of calibration using the le Cessie-van Houwelingen goodness-of-fit test, and the level of discrimination using the concordance (or *C*) statistic [31]. Bias-corrected estimates of the *C* statistic were produced to account for model overfitting [32]. This internal validation consisted of 9,999 bootstrap resamples. Odds ratios (ORs) were calculated for the immune parameters in each model. The analysis was performed using the statistical software packages SPSS (IBM, New York, United States), R version 3.3.2 (http://www.r-project.org) together with the *ggplot2*, *effects*, and *rms* packages, and GraphPad Prism software (GraphPad Software, California, US) on data that were available for each given time point. The threshold for significance was considered to be *p* ≤ 0.05, with nominal *p*-values reported with no adjustment for multiple testing unless otherwise stated. In all figures, the horizontal line displayed in the data points collected from HCs depicts the median value.

## Results

### Patient enrolment and demographics

**Fig 1:**
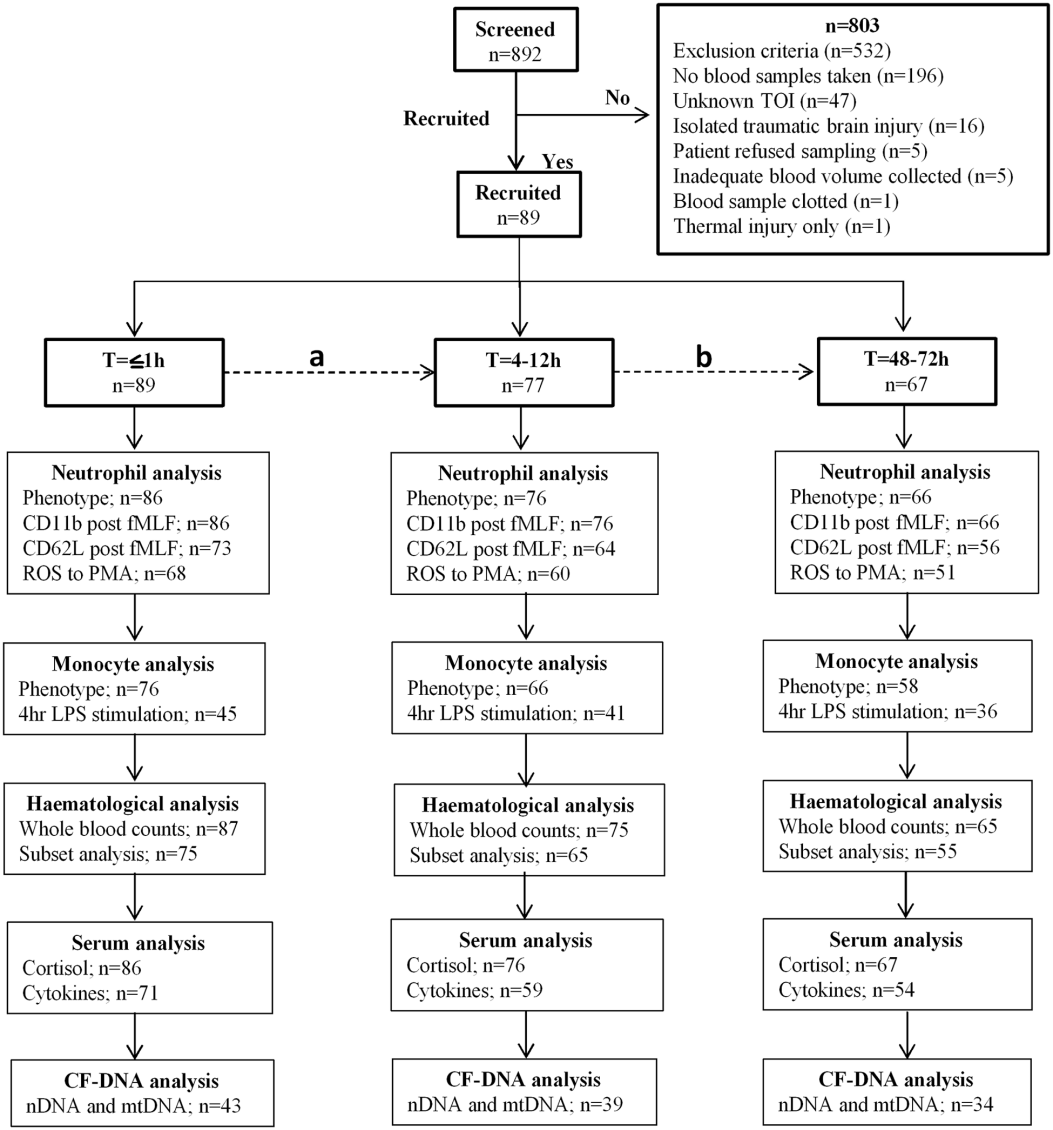
Flow diagram showing recruitment and analysis of study subjects. (A) A total of 12 patients were lost between the T≤1-hour and T = 4–12-hour time points as a result of steroid treatment (*n* = 4), refusal of sampling (*n* = 6), hospital discharge (*n* = 1), and mortality (*n* = 1). (B) Patients lost as a result of mortality (*n* = 2), difficulty in bleeding (*n* = 3), refusal of sampling (*n* = 5), hospital discharge (*n* = 2), and steroid treatment (*n* = 1). Three patients who refused blood sampling at the T = 4–12-hour time point provided samples at the T = 48–72-hour time point. Thus, a total of 10 patients were lost between the T = 4–12-hour and T = 48–72-hour time points. Insufficient sample volume and equipment breakdown account for the differences in patient numbers between each parameter analysed. CF-DNA, cell-free DNA; fMLF, formyl-methionine-leucine-phenylalanine; LPS, lipopolysaccharide; mtDNA, mitochondrial DNA; nDNA, nuclear DNA; PMA, phorbol 12-myristate 13-acetate; ROS, reactive oxygen species; TOI, time of injury.

Fig 1 shows a flow diagram of patient enrolment and sampling. A total of 892 adult trauma patients were screened for inclusion into the study. Of these, 89 patients (mean age 41 years, range 18–90 years, 75 males) with a mean ISS of 24 (range 9–66) were enrolled prospectively (Table 1), with blood samples acquired from all patients ≤1 hour postinjury (mean time to sample 42 minutes, range 17–60 minutes).

**Table 1:** Patient demographics.

	Patients (*n* = 89)	MODS (*n* = 40)	No MODS (*n* = 37)	*p*-Value^¶^
Age, years	41 (18–90)	45 (18–90)	39 (18–79)	NS
Male, *n* (%)	75 (84)	34 (85)	32 (87)	NS
Time to prehospital sample, minutes postinjury	42 (17–60)	43 (17–60)	43 (18–60)	NS
ISS	24 (9–66)	31 (9–66)	18 (9–50)	**0.0001**
NISS	35 (9–75)	44 (9–75)	26 (9–66)	**0.0003**
**AIS**				
Head, n (%)	42 (47)	30 (86)	10 (29)	<**0.0001**
Face/ Neck, *n* (%)	31 (34)	18 (51)	9 (26)	**0.03**
Thorax, *n* (%)	50 (56)	22 (63)	22 (63)	NS
Abdomen, *n* (%)	23 (26)	10 (29)	11 (31)	NS
Spine, *n* (%)	26 (29)	19 (54)	6 (17)	**0.001**
Upper extremities, *n* (%)	34 (38)	17 (49)	16 (46)	NS
Lower extremities, *n* (%)	33 (37)	16 (46)	17 (49)	NS
Admission GCS score	10 (3–15)	8 (3–15)	13(3–15)	<**0.001**
Lactate mmol/L^δ^	4.43 (0.5–14.9)	4.44 (0.5–14.9)	4.04 (1.6–14.7)	NS
Base Excess mmol/L^δ^	−4.33 (−16.80 to 2.10)	−5.75 (−16.80 to 2.1)	−2.65 (−16.30 to 1.4)	**0.0002**
**Mechanism of injury**				
Fall, *n* (%)	15 (17)	8 (20)	7 (19)	NS
A/P, *n* (%)	21 (24)	3 (8)	11 (30)	**0.01**
Blunt, *n* (%)	3 (3)	0 (0)	2 (5)	NS
RTC, *n* (%)	50 (56)	29 (73)	17 (46)	**0.01**
**Outcomes**				
ICU-free days	23 (0–30)	17 (0–30)	26 (9–30)	<**0.001**
Hospital-free days	13 (0–29)	7 (0–26)	15 (0–26)	**0.0007**
Mortality, *n* (%)	13 (15)	10 (25)	0 (0)	**0.001**

^¶^Comparison of MODS versus No MODS.

^δ^Values obtained from first post-hospital admission readings.

Data are expressed as mean (range) unless indicated otherwise. For all patients (*n* = 89), the number of data points for each clinical variable are as follows: ISS, *n* = 79; NISS, *n* = 75; AIS scores, *n* = 77, GCS, *n* = 89; and Lactate and Base Excess, *n* = 83. For MODS patients (*n* = 40), the number of data points for each clinical variable are as follows: ISS, *n* = 36; NISS, *n* = 34; AIS scores, *n* = 35, GCS, *n* = 40; and Lactate and Base Excess, *n* = 38. For No MODS patients (*n* = 37), the number of data points for each clinical variable are as follows: ISS, *n* = 35; NISS, *n* = 34; AIS scores, *n* = 35, GCS, *n* = 37; and Lactate and Base Excess, *n* = 35. A/P, assault/penetrating; AIS, abbreviated injury scale; GCS, Glasgow coma scale; ISS, injury severity score; ICU, intensive care unit; LOS, length of stay; MODS, multiple organ dysfunction syndrome; NISS, new injury severity score; NS, nonsignificant; RTC, road traffic collision.

### Traumatic injury results in an immediate and persistent leukocytosis

Analysis of whole blood cell counts revealed a significant leukocytosis within minutes of traumatic injury that remained at the 4–12- and 48–72-hour time points (Table 2). Underlying the immediate leukocytosis were significant elevations in monocyte, neutrophil, IG, and lymphocyte counts (Table 2), with the lymphocytosis driven by a significant increase in the absolute number of B cells, NK cells, NKT cells, and both CD4^+^ and CD8^+^ T cells (Table 3). Further phenotypical analysis of lymphocyte subsets revealed an immediate post-trauma elevation in the numbers of CD56^DIM^ cytotoxic NK cells as well as CD4^+^ and CD8^+^ effector memory T cells and CD4^+^ and CD8^+^ central memory subsets (Table 3). For CD8^+^ T cells, a significant increase in highly differentiated TEMRA cells was also seen (Table 3).

**Table 2:** Whole blood cell counts and percentages.

	HCs (*n* = 30)	≤1 hour (*n* = 87)	4–12 hours (*n* = 75)	48–72 hours (*n* = 65)
**Counts (10**^**9**^**/L)**				
WBCs	5.66 ± 0.21	13.76 ± 0.60***	18.31 ± 0.80***	11.98 ± 0.73***
Neutrophils	3.11 ± 0.16	8.34 ± 0.50***	15.67 ± 0.73***	9.51 ± 0.67***
Lymphocytes	1.87 ± 0.09	4.28 ± 0.20***	1.23 ± 0.06***	1.32 ± 0.06*
Monocytes	0.45 ± 0.02	0.84 ± 0.04***	1.35 ± 0.08***	0.84 ± 0.06***
IGs	0.013 ±0.001	0.218 ± 0.027***	0.131 ± 0.017***	0.084 ± 0.021***
**Percentages**				
Neutrophils	54.48 ± 1.41	58.02 ± 1.47	85.00 ± 0.56***	77.48 ± 1.14***
Lymphocytes	33.47 ± 1.14	33.44 ± 1.40	7.29 ± 0.42***	12.62 ± 0.79***
Monocytes	8.18 ± 0.34	6.39 ± 0.23***	7.40 ± 0.27	7.22 ± 0.30
IGs	0.27 ± 0.02	1.43 ± 0.15***	0.62 ± 0.05***	0.55 ± 0.06**

*p < 0.05 versus HCs,

**p < 0.005 versus HCs,

***p < 0.0005 versus HCs.

Data are presented as mean ± SEM. HC, healthy control; IG, immature granulocyte; WBC, white blood cell.

**Table 3:** Absolute numbers (10^6^/L) of lymphocyte subsets.

	HCs (*n* = 19)	≤1 hour	4–12 hours	48–72 hours
**B cells**	135 ± 12	314 ± 28**	149 ± 17	184 ± 18
**NK cells**	319 ± 34	1,014 ± 78**	202 ± 24	132 ± 17***
CD3^−^56^DIM^	301 ± 33	983 ± 77**	196 ± 23	126 ± 17***
CD3^−^56^BRIGHT^	18 ± 3	31 ± 3	6 ± 1***	6 ± 1***
**NKT cells**	65 ± 12	311 ± 51*	41 ± 6	60 ± 11
**CD3**^**+**^	1297 ± 120	2,704 ± 147**	747 ± 53*	877 ± 60
**CD3**^**+**^**4**^**+**^	759 ± 79	1407 ± 78*	457 ± 34*	543 ± 42
TEMRA	29 ± 4	79 ± 12	12 ± 2**	17 ± 2
Naïve	423 ± 66	594 ± 45	214 ± 21**	252 ± 23
Central Memory	155 ± 19	326 ± 23**	139 ± 11	158 ± 15
Effector Memory	152 ± 11	408 ± 41*	92 ± 8*	116 ± 13
**CD3**^**+**^**8**^**+**^	424 ± 65	1,146 ± 99***	253 ± 23	295 ± 27
TEMRA	179 ± 44	518 ± 69*	70 ± 11*	96 ± 15
Naïve	158 ± 28	250 ± 29	114 ± 17	111 ± 15
Central Memory	9 ± 3	39 ± 5***	15 ± 2	22 ± 6
Effector Memory	78 ± 11	339 ± 34***	54 ± 6	66 ± 7

*p < 0.05 versus HCs,

**p < 0.005 versus HCs,

***p < 0.0005 versus HCs.

Data are presented as mean ± SEM. B cells, ≤1 hour, *n* = 72; 4–12 hours, *n* = 62; 48–72 hours, *n* = 53. NK and NKT cells, ≤1 hour, *n* = 70; 4–12 hours, *n* = 59; 48–72 hours, *n* = 51. CD3^+^ cells, ≤1 hour, *n* = 75; 4–12 hours, *n* = 65; 48–72 hours, *n* = 55. HC, healthy control; NK, natural killer; NKT, natural killer T; TEMRA, terminally differentiated effector memory.

In the acute postinjury phase, a significant bifurcation was seen in the innate and adaptive immune cell responses. The monocytosis, neutrophilia, and elevated IG counts persisted at the 4–12- and 48–72-hour postinjury time points, whereas the lymphocyte counts were significantly lower than the values for HCs. This trauma-induced lymphopenia was comprehensive with reduced numbers of CD56^DIM^ and CD56^BRIGHT^ NK cells, TEMRA CD4^+^ and CD8^+^ T cells, and both naive and effector memory CD4^+^ T cells (Table 3).

### Neutrophil ROS production

**Fig 2:**
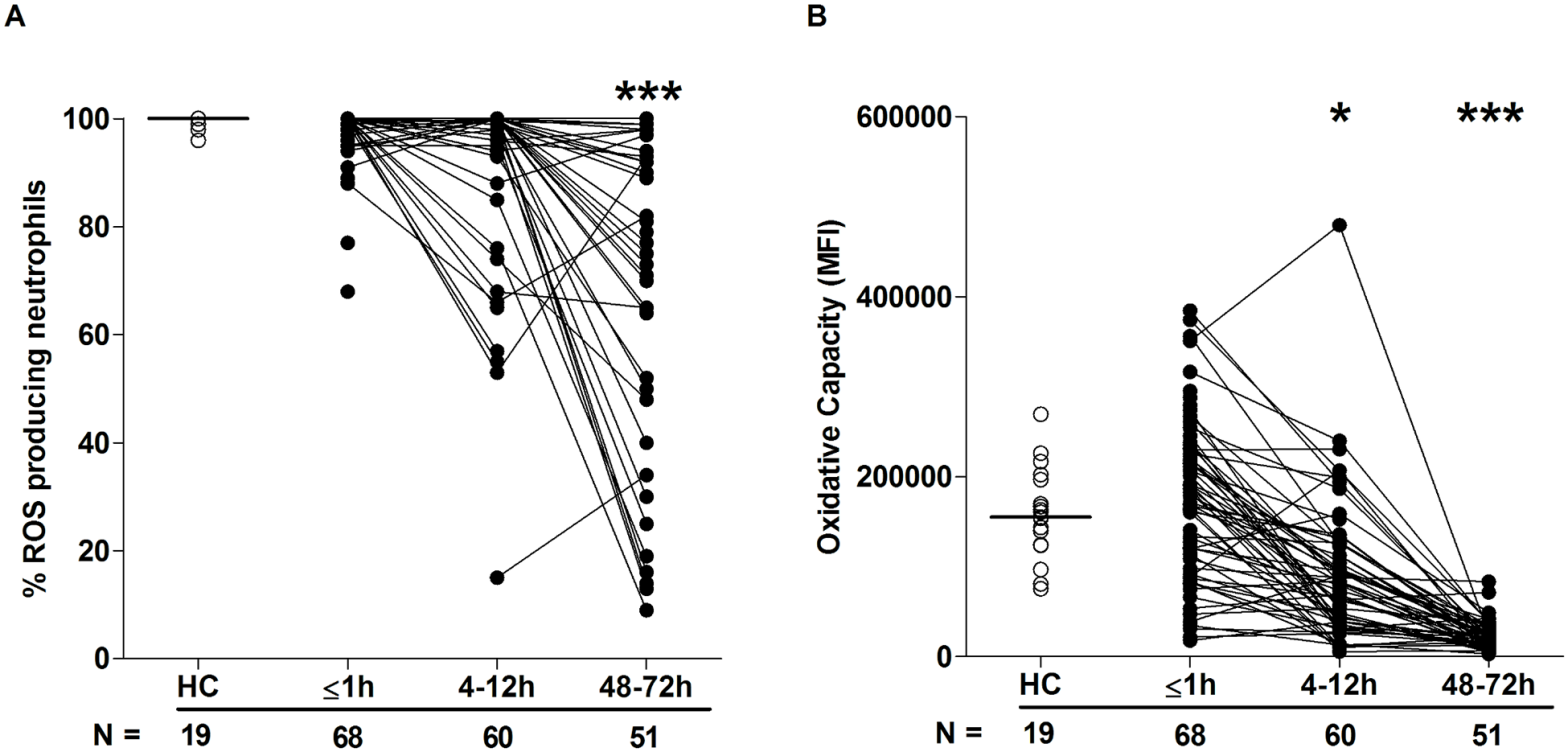
Neutrophil ROS production postinjury. (A–B) Neutrophil ROS generation in response to PMA stimulation was assessed across time post-trauma. Data are presented as (A) percentage of neutrophils that produced ROS and (B) oxidative capacity. The number of samples analysed is indicated below each time point. The horizontal line for HC data depicts the median value. *p < 0.05, ***p < 0.0005 versus HCs. HC, healthy control; MFI, mean fluorescence intensity; PMA, phorbol 12-myristate 13-acetate; ROS, reactive oxygen species.

In response to stimulation with the protein kinase C activator PMA, ROS production by neutrophils isolated from trauma patients within minutes of injury was comparable to that recorded for HCs (Fig 2A and 2B). However, at the 4–12-hour and 48–72-hour time points, a significant reduction in both the percentage of ROS producing neutrophils (Fig 2A) and the oxidative capacity of each cell was observed (Fig 2B).

### Sterile traumatic injury triggers the immediate release of nuclear DNA with evidence of neutrophil extracellular trap (NET) generation

**Fig 3:**
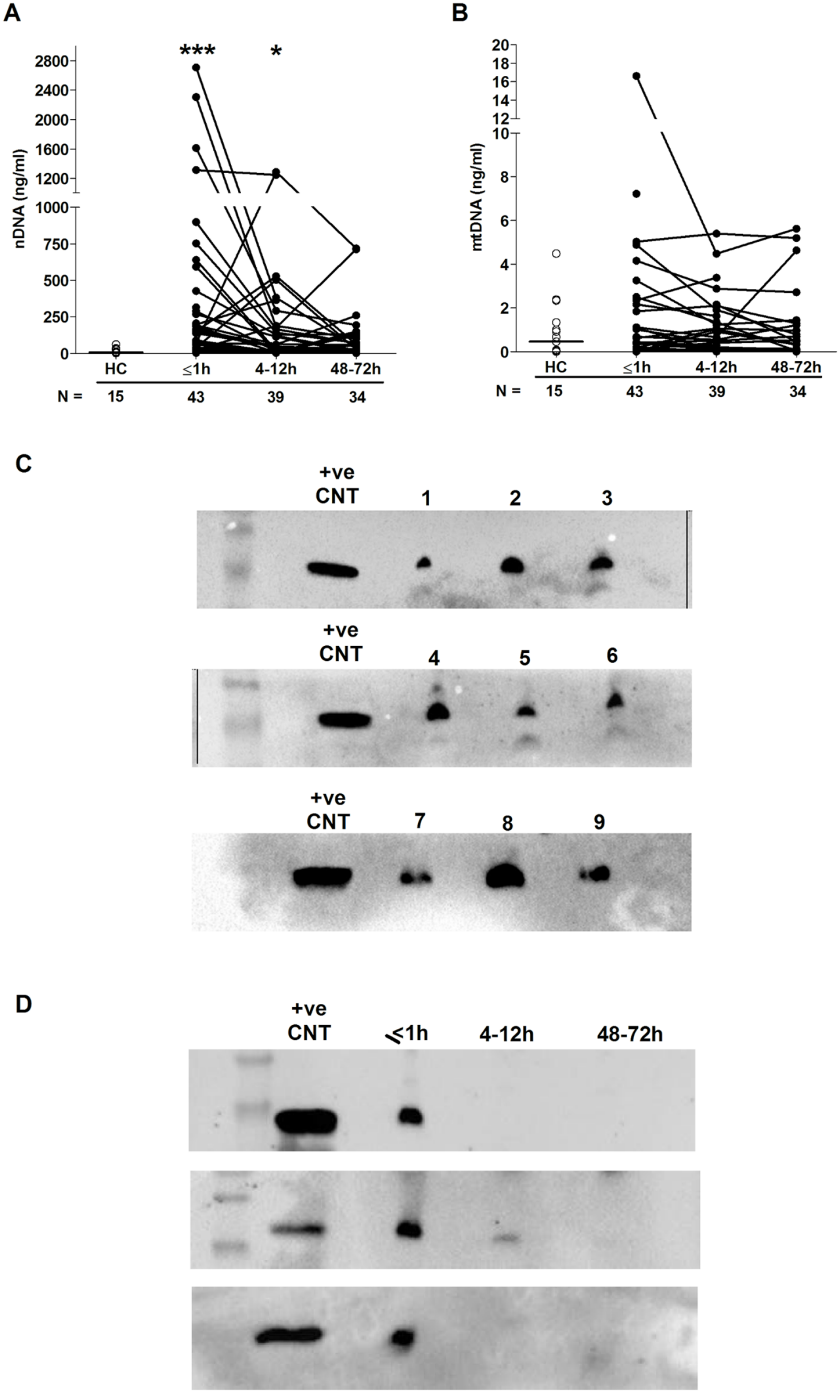
Evidence of NET formation during the ultra-early immune response to sterile traumatic injury. (A–B) Plasma concentrations (ng/ml) of nuclear (A) and mitochondrial (B) DNA across time post-trauma. The number of samples analysed is indicated below each time point. The horizontal line for HC data depicts the median value. *p < 0.05, ***p < 0.0005 versus HCs. (C–D) Western blots showing the levels of citrullinated histone H3 in plasma samples obtained from 9 trauma patients within 1 hour of injury (C) and in 3 trauma patients across the 3 postinjury time points (D). +ve CNT, positive control; HC, healthy control; mtDNA, mitochondrial DNA; nDNA, nuclear DNA; NET, neutrophil extracellular trap.

Relative to HCs, we detected significantly elevated concentrations of nuclear but not mitochondrial DNA in plasma samples collected ≤1 hour and 4–12 hours postinjury (Fig 3A and 3B). We hypothesised that 1 possible source of this nuclear DNA was neutrophils via their production of extracellular traps, a defence mechanism in which neutrophils extrude their DNA into the extracellular environment. To test this, we screened plasma samples for CitH3, a protein that decorates the nuclear DNA backbone of NETs [33]. Western blotting revealed the presence of CitH3 in samples acquired within 1 hour of injury, but not in those obtained at the 4–12- or 48–72-hour postinjury time points (Fig 3C and 3D).

### Neutrophil surface phenotype and responsiveness to damage-associated molecular pattern (DAMP) stimulation following injury

To study the activation status of neutrophils ex vivo, we measured, in the absence of external stimulation, the surface density of the adhesion molecules CD62L and CD11b as well as the chemokine receptors CXCR1 and CXCR2. Compared to values for HCs, CD11b and CXCR2 surface expression was significantly increased and decreased, respectively, on neutrophils isolated from blood samples acquired within 1 hour of injury, with no differences detected for CD62L or CXCR1 expression (Table 4). At the 4–12- and 48–72-hour time points, neutrophils exhibited significantly reduced CD62L and CXCR2 expression, which was accompanied by increased CD11b expression (Table 4). CXCR1 surface expression was significantly reduced relative to HC values only at the 4–12-hour postinjury time point (Table 4). An assessment of CD88 expression, the receptor for the complement factor C5a, revealed a significant trauma-induced reduction in surface density 48–72 hours postinjury (Table 4).

**Table 4:** Neutrophil surface phenotype.

	**HCs (*n* = 17)**	**≤1 hour (*n* = 86)**	**4–12 hours (*n* = 76)**	**48–72 hours (*n* = 66)**
**CD11b**	8,076 ± 1,246	26,071 ± 2,114***	17,169 ± 894***	15,110 ± 1,780*
**CD16**	107,536 ± 9,669	145,651 ± 5,916*	100,891 ± 5,080	95,159 ± 5,918
**CD62L**^#^	34,270 ± 1,646	29,224 ± 1,067	26,198 ± 1,069***	24,980 ± 1,019***
**CD63**	2,095 ± 629	2,258 ± 228	1,148 ± 94	2,473 ± 235
**CD88**	97,881 ± 6,212	78,596 ± 2,623	82,339 ± 3,147	56,987 ± 2,597***
**CXCR1**^¶^	17,458 ± 571	16,552 ± 472	14,343 ± 418**	20,544 ± 616
**CXCR2**^$^^,^^¶^	28,071 ± 1,822	19,391 ± 934***	15,945 ± 739***	21,347 ± 966*
**CD11b post-fMLF**	73,200 ± 5,429	87,009 ± 3,340	82,150 ± 2,610	62,231 ± 2,414
**Fold increase in CD11b post-fMLF**	10.95 ± 1.04	4.46 ± 0.27***	5.54 ± 0.27***	5.60 ± 0.38***
	**HCs (*n* = 16)**	**≤1 hour (*n* = 73)**	**4–12 hours (*n* = 64)**	**48–72 hours (*n* = 56)**
**CD62L post-fMLF**	1,775 ± 207	4,289 ± 353***	3,330 ± 392	3,069 ± 287
**Fold decrease in CD62L post-fMLF**	22.90 ± 2.69	9.67 ± 0.69***	14.97 ± 2.26**	12.14 ± 1.18***

*p < 0.05 versus HC,

**p < 0.005 versus HC,

***p < 0.0005 versus HC.

^#^CD62L for 4–12 hours, *n* = 75.

^$^CXCR2 for ≤1 hour, *n* = 85.

^¶^CXCR1 and CXCR2 for HC, *n* = 16.

Data are presented as mean ± SEM. fMLF, formyl-methionine-leucine-phenylalanine; HC, healthy control.

**Fig 4:**
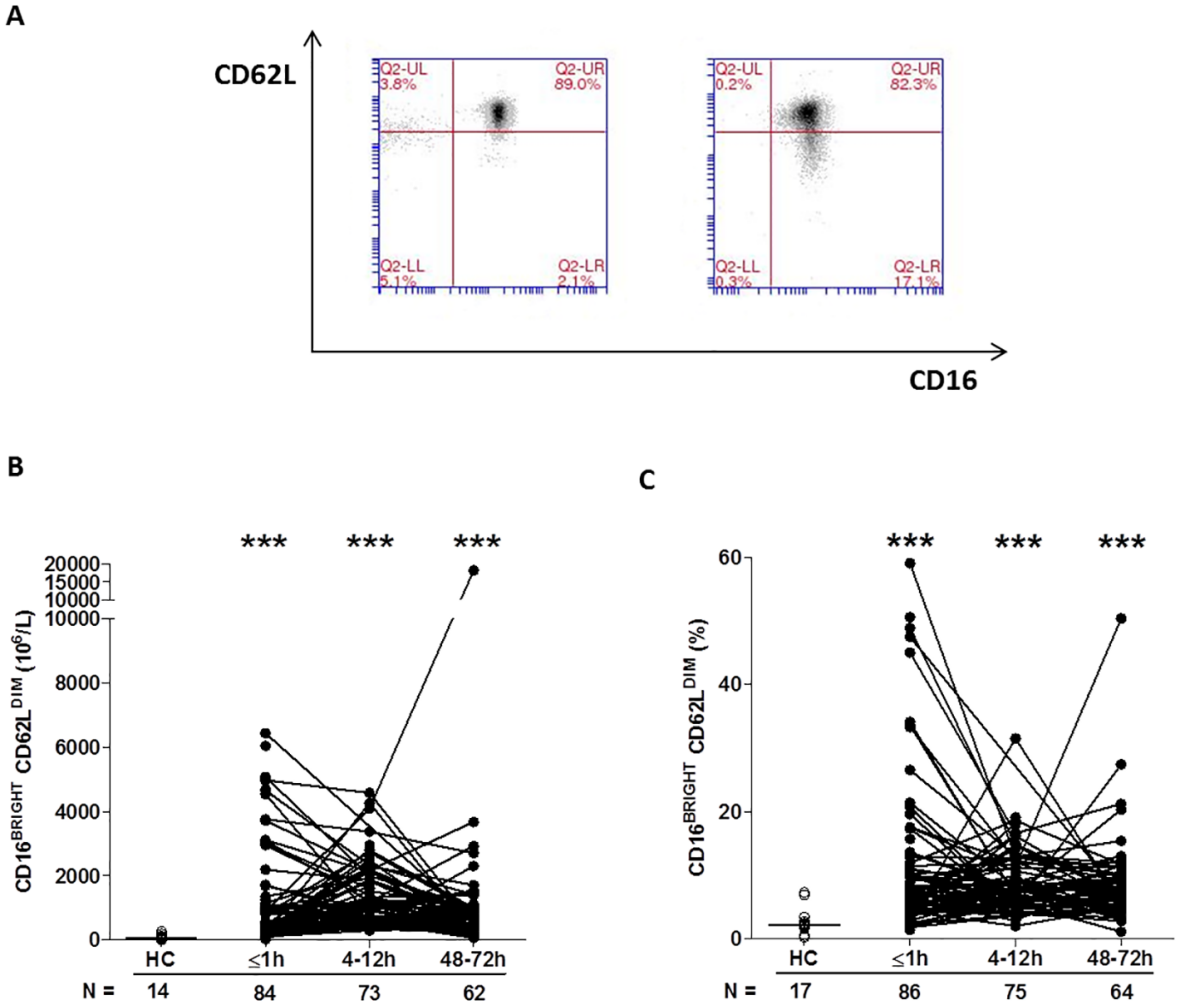
Traumatic injury results in immediate alterations in the composition of the circulating neutrophil pool. (A) Flow cytometry plots depicting the percentage of CD16^BRIGHT^ CD62L^DIM^ neutrophils (lower right quadrant) in blood samples from a single healthy control (left panel) and a trauma patient (right panel). (B–C) Prospective analysis of the absolute number (B) and frequency (C) of circulating CD16^BRIGHT^ CD62L^DIM^ neutrophils post-trauma. The number of samples analysed is indicated below each time point. ***p < 0.0005 versus HCs. The horizontal line for HC data depicts the median value. HC, healthy control.

By combining CD16 expression data with our analysis of CD62L, we could determine the presence of CD16^BRIGHT^ CD62L^DIM^ neutrophils, a highly mature neutrophil subset with immunosuppressive properties [34]. In samples collected within 1 hour of trauma, as well as 4–12 and 48–72 hours postinjury, we found a significantly increased frequency and absolute number of CD16^BRIGHT^ CD62L^DIM^ neutrophils relative to the values recorded for HCs (Fig 4A–4C).

To examine the responsiveness of neutrophils to agents released as a result of tissue injury—so-called damage-associated molecular patterns (DAMPs)—we measured CD11b and CD62L surface density following 5 minutes of stimulation with the formylated peptide fMLF. Relative to baseline readings, the fold increase in CD11b expression that occurred post-fMLF stimulation was significantly lower for neutrophils isolated from trauma patients within minutes of injury as well as 4–12 and 48–72 hours postinjury when compared to HCs (Table 4). At all 3 postinjury time points, neutrophils from trauma patients shed significantly less CD62L following fMLF stimulation when compared to neutrophils from HCs (Table 4).

### Traumatic injury is associated with immediate alterations in LPS-induced cytokine production by whole blood leukocytes that persist into the acute postinjury phase

Analysis of blood samples acquired within 1 hour of injury revealed an immediate impairment in LPS-induced cytokine production by whole blood leukocytes. Relative to HCs, significantly lower concentrations of IL-6, TNF-α, and MCP-1 were present in supernatants collected from blood samples obtained within minutes of injury following a 4-hour stimulation with 1 or 10 ng/ml LPS (Table 5). In addition, we detected a significant reduction in IL-10 secretion by leukocytes when challenged with 1 ng/ml LPS (Table 5). However, traumatic injury had no immediate impact upon LPS-induced IL-8 production (Table 5). With the exception of IL-10 secretion in response to 10 ng/ml LPS, significantly lower concentrations of IL-6, IL-8, IL-10, TNF-α, and MCP-1 were detected in supernatants of blood samples acquired from patients 4–12 hours postinjury following stimulation with 1 or 10 ng/ml LPS (Table 5). At the 48–72-hour postinjury time point, significant trauma-induced impairments in LPS-stimulated cytokine production were recorded only for IL-6, IL-10, and TNF-α (Table 5).

**Table 5:** Cytokine and chemokine concentrations measured in whole blood following 4-hour stimulation with 1 or 10 ng/ml LPS.

	HCs (*n* = 14)	≤1 hour (*n* = 45)	4–12 hours (*n* = 41)	48–72 hours^#^ (*n* = 36)
**IL-6**				
1 ng/ml LPS	47,294 ± 6,681	12,184 ± 1,386***	6,932 ± 1,291***	14,791 ± 2,416***
10 ng/ml LPS	83,531 ± 10,630	26,327 ± 2,570***	19,267 ± 3,584***	31,618 ± 3,963**
**TNF-α**				
1 ng/ml LPS	21,927 ± 2,675	2,900 ± 471***	1,464 ± 278***	3,829 ± 616***
10 ng/ml LPS	39,431 ± 4,001	6,236 ± 765***	4,365 ± 971***	8,887 ± 1,330***
**MCP-1**				
1 ng/ml LPS	2,756 ± 257	1,694 ± 132*	1,469 ± 160***	2,452 ± 256
10 ng/ml LPS	3,106 ± 316	2,004 ± 144*	1,789 ± 172**	2,904 ± 309
**IL-10**				
1 ng/ml LPS	267 ± 60	225 ± 94*	183 ± 46*	230 ± 133***
10 ng/ml LPS	328 ± 67	278 ± 86	261 ± 49	284 ± 146**
**IL-8**				
1 ng/ml LPS	6,030 ± 925	5,502 ± 723	3,273 ± 471*	6,281 ± 1,069
10 ng/ml LPS	10,697 ± 1,527	8,884 ± 1,011	6,539 ± 903*	9,609 ± 1,190

*p < 0.05 versus HCs,

**p < 0.005 versus HCs,

***p < 0.0005 versus HCs.

^#^For 10 ng/ml LPS stimulations, *n* = 34 for the 48–72-hour time point.

Data are presented as mean ± SEM. HC, healthy control; IL, interleukin; LPS, lipopolysaccharide; MCP-1, monocyte chemoattractant protein-1; TNF-α, tumour necrosis factor-alpha.

### Changes to the surface phenotype of monocytes are evident within minutes of injury

Categorising monocytes as CD14^++^16^−^ or CD14^++^16^+^, we found—in blood samples collected within 1 hour of injury—a significant increase, relative to HCs, in the absolute number of both subsets (Table 6). This trauma-induced elevation persisted 4–12 and 48–72 hours postinjury, with a significantly increased and decreased frequency of CD14^++^16^+^ and CD14^++^16^−^ monocytes, respectively, observed at the latter time point (Table 6).

**Table 6:** Monocyte subsets and surface phenotype.

	HCs	≤1 hour	4–12 hours	48–72 hours
**CD14**^**+**^**16**^**−**^				
*n*	26	75	65	58
%	91.81 ± 1.32	90.23 ± 0.72	92.78 ± 0.70	86.73 ± 0.90**
*n*	23	73	63	56
Number (10^6^/L)	410 ± 21	802 ± 48***	1,302 ± 86***	730 ± 62**
**CD14**^**+**^**16**^**+**^				
*n*	26	75	65	58
*%*	8.14 ± 1.32	9.76 ± 0.72	7.21 ± 0.70	13.27 ± 0.90**
*n*	23	73	63	56
Number (10^6^/L)	42 ± 9	87 ± 9**	98 ± 12**	121 ± 16***
**CD16**				
*n*	26	75	65	58
% Positive	9.5 ± 1.2	10.4 ± 0.8	8.2 ± 0.7	14.3 ± 0.9**
MedFI	0.8 ± 0.2	1.3 ± 0.2	0.6 ± 0.1	0.8 ± 0.1
**HLA-DR**				
*n*	26	76	66	58
% Positive	99.3 ± 0.2	98.0 ± 0.3	95.4 ± 0.5***	84.0 ± 1.5***
MedFI	225 ± 15	173 ± 9	90 ± 5***	49 ± 4***
**TLR2**				
*n*	18	71	61	54
% Positive	99.9 ± 0.06	99.8 ± 0.06	99.9 ± 0.04	99.9 ± 0.02
MedFI	165 ± 9	251 ± 10***	181 ± 8	210 ± 10
**TLR4**				
*n*	26	72	61	52
% Positive	34.9 ± 3.1	49.5 ± 1.9**	35.9 ± 1.9	45.7 ± 2.1*
MedFI	6 ± 0.5	9 ± 0.7***	7 ± 0.3	8 ± 0.4**
**CD86**				
*n*	25	71	61	52
% Positive	99.9 ± 0.04	99.5 ± 0.1	94.1 ± 1.6***	94.1 ± 0.9***
MedFI	53 ± 4	75 ± 3***	41 ± 2	45 ± 2

*p < 0.05,

**p < 0.005,

***p < 0.0005 versus HCs.

Data are presented as mean ± SEM. HC, healthy control; HLA-DR, human leukocyte antigen DR; MedFI, median fluorescence intensity; TLR, Toll-like receptor.

Surface expression of the antigen-presenting molecule human leukocyte antigen DR (HLA-DR) is commonly used as a marker of the immune competence of circulating monocytes. When measured as either the percentage of positive cells or surface density, we detected no difference in HLA-DR expression between monocytes isolated from trauma patients within minutes of injury and HCs (Table 6). However, at the 4–12- and 48–72-hour time points, a significant trauma-induced reduction in both parameters of HLA-DR expression was observed (Table 6).

**Fig 5:**
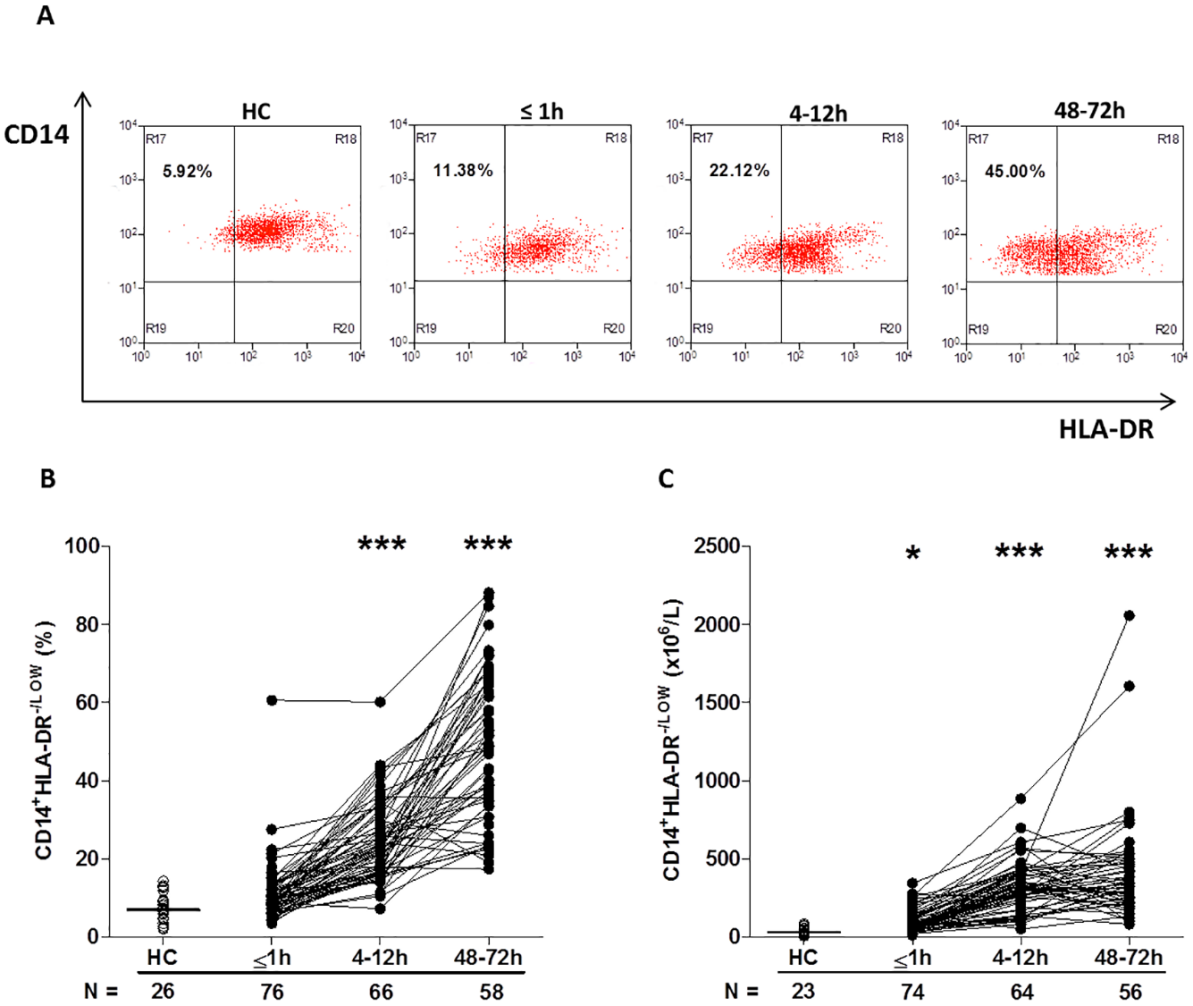
Traumatic injury results in elevated percentages and absolute numbers of circulating CD14^+^HLA-DR^-/low^ immunosuppressive monocytes. (A) Representative flow cytometry plots depicting the percentage of CD14^+^HLA-DR^-/low^ monocytes (upper left quadrant) in a single HC and a trauma patient across time. (B–C) Prospective assessment of the percentage (B) and absolute number (C) of CD14^+^HLA-DR^-/low^ monocytes post-trauma. The number of samples analysed is indicated below each time point. The horizontal line for HC data depicts the median value. *p < 0.05, ***p < 0.0005 versus HC. HC, healthy control.

Furthermore, compared to the values recorded for HCs, trauma patients presented in the immediate aftermath of injury with a significantly higher absolute number of CD14^+^HLA-DR^low/−^ monocytes (−41; 95% confidence interval [CI] −61.00 to −25.00; *p* < 0.001, Fig 5C), a subset that has been shown to have immune suppressive properties [35]. Elevated counts remained at the 4–12-hour (−281; 95% CI −329 to −246; *p* < 0.001) and 48–72-hour (−308; 95% CI −380 to −244; *p* < 0.001) time points, when significantly increased frequencies of CD14^+^HLA-DR^low/−^ monocytes were also observed (Fig 5A–5C).

Analysis of TLR expression revealed an immediate and significant trauma-induced increase in the surface density of TLR2 and TLR4, which in the case of TLR4 was accompanied by an increased percentage of receptor positive monocytes (Table 6). In subsequent 4–12-hour samples, both TLR2 and TLR4 expression returned to a level comparable to HCs (Table 6). Whilst this remained the case for TLR2 at the 48–72-hour time point, a significant increase in both the percentage of TLR4^+^ monocytes and TLR4 surface density was found (Table 6).

Compared to the values of HCs, we found surface density of the costimulatory molecule CD86 was significantly higher on monocytes isolated from trauma patients within 1 hour of injury (Table 6). At the 4–12- and 48–72-hour postinjury time points, CD86 surface density returned to a level comparable to that of HCs, although the frequency of CD86^+^ monocytes was significantly lower (Table 6).

### Traumatic injury results in an immediate and sustained systemic inflammatory response

**Fig 6:**
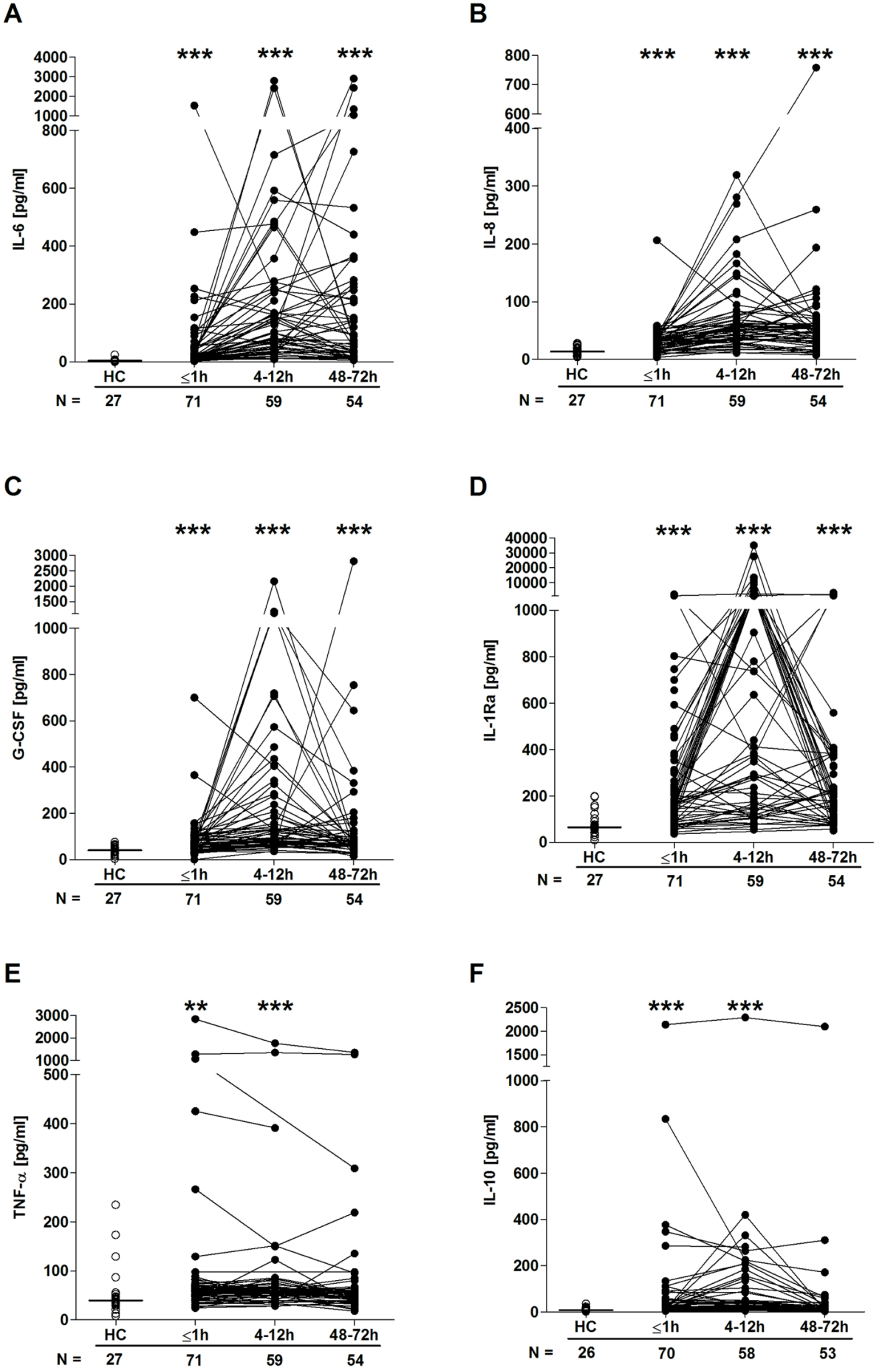
Serum cytokine and chemokine concentrations post-trauma. Serum concentrations of IL-6 (A), IL-8 (B), G-CSF (C), IL-1Ra (D), TNF-α (E), and IL-10 (F) across time post-trauma. The number of patient and HC samples analysed is indicated below each time point. The horizontal line for HC data depicts the median value. **p < 0.005, ***p < 0.0005 versus HCs. G-CSF, granulocyte-colony stimulating factor; HC, healthy control; IL, interleukin; IL-1Ra, interleukin-1 receptor antagonist; TNF- α, tumour necrosis factor-alpha.

**Fig 7:**
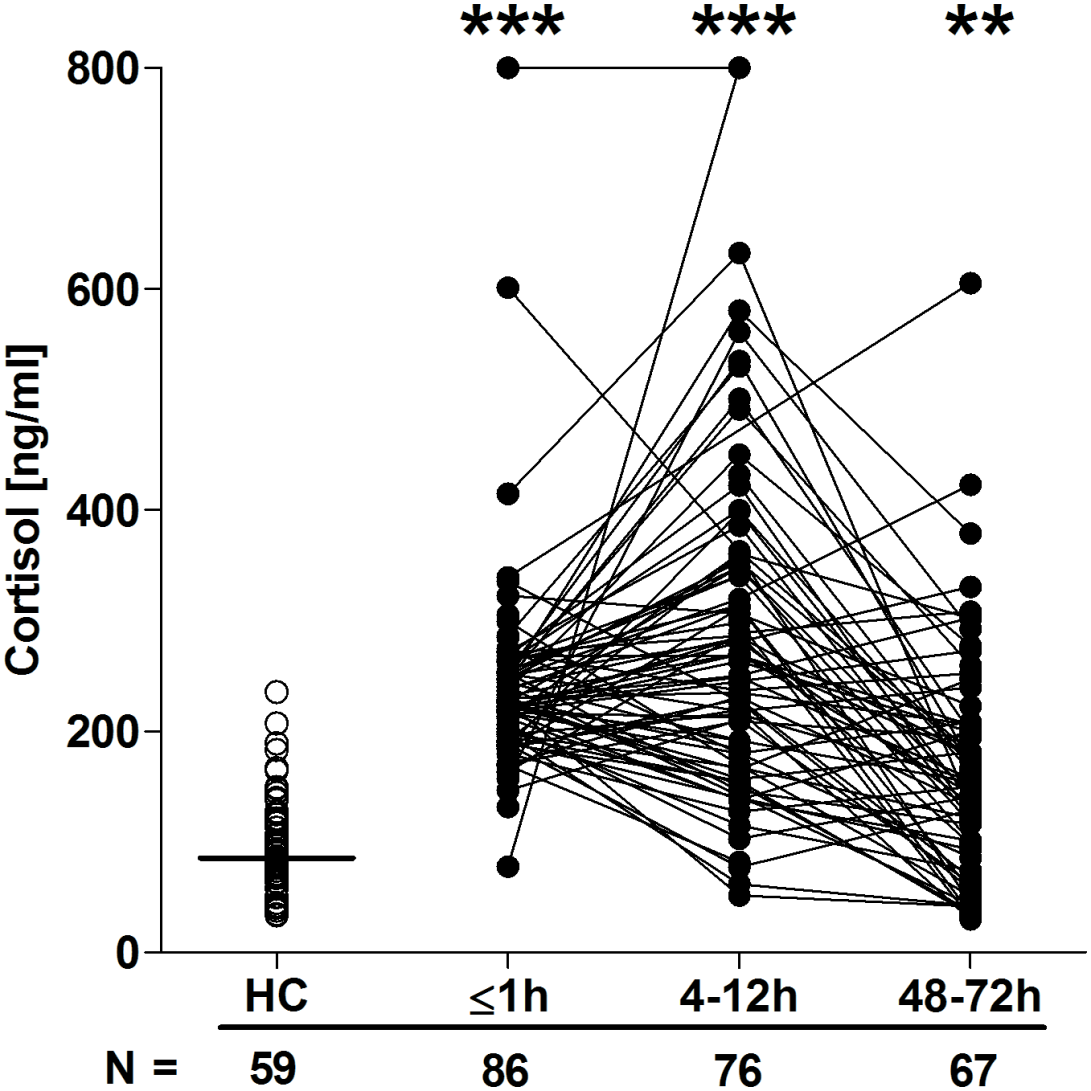
Serum cortisol concentrations post-trauma. The number of patient and HC samples analysed is indicated below each time point. The horizontal line for HC data depicts the median value. **p < 0.005, ***p < 0.0005 versus HCs. HC, healthy control.

Quantification of cytokine and chemokine concentrations in patient serum samples revealed an immediate postinjury elevation in pro- and anti-inflammatory mediators. Relative to HCs, concentrations of IL-6, IL-8, G-CSF, IL1-Ra, TNF-α, and IL-10 were all significantly greater in blood samples acquired in the immediate aftermath of trauma, with these elevations persisting at the 4–12-hour time point (Fig 6A–6F). By 48–72 hours postinjury, whilst serum IL-10 and TNF-α concentrations had returned to levels seen in HCs, IL-6, IL-8, G-CSF, and IL1-Ra levels remained significantly increased (Fig 6A–6F). At no postinjury time point did we observe any significant difference in serum MCP-1 concentrations between patients and HCs (S1 Fig). An assessment of serum cortisol levels revealed a significant trauma-induced increase in circulating concentrations at all 3 postinjury time points when compared to HCs (Fig 7).

### Specific immune responses in the prehospital setting are associated with the development of MODS

As previous analysis of post-hospital admission blood samples had revealed that trauma-induced alterations in lymphocyte number and monocyte function/phenotype were associated with the development of MODS [9,36,37], we performed an exploratory data analysis to examine whether, in samples acquired within minutes of injury, any potential associations existed between these facets of the immune response or other measures of immune activation and the development of MODS. From our cohort of 89 patients, 12 were excluded from analysis due to either mortality (*n* = 3) or hospital discharge (*n* = 9) within 72 hours of injury, as a length of stay (LOS) ≥ 4 days was required for patients to meet the definition of MODS adopted in this study (S2 Fig). Of the 77 remaining patients, 40 (52%) developed MODS (Table 1). A higher incidence of head, face/neck, and spinal injuries was present amongst patients in the MODS group, who also exhibited significantly higher ISS and NISS as well as lower admission Glasgow coma scale (GCS) scores and base excess (an indicator of haemorrhagic shock) when compared to those patients who did not develop MODS (Table 1). In terms of outcomes, both ICU and hospital-free days were significantly lower for MODS patients, in whom a higher incidence of mortality was also recorded (Table 1).

For all immune and inflammatory parameters assessed (Tables 7 and 8), only NKT and CD3^+^8^+^ cell numbers were found to be significantly different between the 2 patient groups after controlling for a false discovery rate of 5%, with elevated values present in samples acquired within 1 hour of injury from those patients who later developed MODS (mean difference of 383, 95% CI 153–614, nominal *p* = 0.0006 for NKT number and mean difference of 509, 95% CI 193–811, nominal *p* = 0.0015 for CD3^+^8^+^; Table 7).

**Table 7:** Comparison of leukocyte counts (10^6^/L) in blood samples acquired within 1 hour of injury between patients who did or did not develop MODS.

Variable	MODS (*n*)	No MODS (*n*)	MODS	No MODS	Nominal *p*-value
Lymphocytes	39	36	4,799 ± 316	3,772 ± 239	0.0125
B cells	27	33	418 ± 58	238 ± 24	0.0134
**NK cells**	27	31	1,102 ± 137	960 ± 104	0.5432
CD3^−^56^DIM^	27	31	1,069 ± 135	934 ± 102	0.5747
CD3^−^56^BRIGHT^	27	31	33 ± 4	25 ± 4	0.054
NKT cells	27	31	540 ± 110	157 ± 25	**0.0006**^#^
CD3^+^	31	32	3,032 ± 240	2,391 ± 182	0.0364
**CD3**^**+**^**4**^**+**^	31	32	1,467 ± 123	1,350 ± 111	0.4838
TEMRA	31	32	97 ± 16	60 ± 18	0.0066
Naive	31	32	515 ± 68	633 ± 71	0.2454
Central Memory	31	32	313 ± 35	345 ± 32	0.4252
Effector Memory	31	32	542 ± 86	313 ± 30	0.0141
**CD3**^**+**^**8**^**+**^	31	32	1,499 ± 198	844 ± 80	**0.0015**^#^
TEMRA	31	32	717 ± 145	348 ± 50	0.0412
Naive	31	32	274 ± 57	211 ± 32	0.8205
Central Memory	31	32	42 ± 9	36 ± 5	0.7517
Effector Memory	31	32	466 ± 64	248 ± 29	0.0097

^#^denotes *p*-values that remain statistically significant after controlling for a false discovery rate of 0.05 using Benjamini and Hochberg’s approach [30].

MODS, multiple organ dysfunction syndrome; NK, natural killer; NKT, natural killer T; TEMRA, terminally differentiated effector memory.

**Table 8:** Comparison of neutrophil and monocyte surface phenotype, monocyte function, and serum cytokines in blood samples acquired within 1 hour of injury from patients who did or did not develop MODS.

Variable	MODS (*n*)	No MODS (*n*)	MODS	No MODS	Nominal *p*-value
**Neutrophil analysis**					
CD11b MedFI	38	36	28,992 ± 3,966	21,033 ± 2,091	0.1713
CD62L MedFI	38	36	30,307 ± 1,717	28,722 ± 1,298	0.2983
CXCR1 MedFI	38	36	15,975 ± 687	17,040 ± 559	0.0591
CXCR2 MedFI	37	36	17,664 ± 1,164	20,488 ± 1,531	0.1238
**Monocyte analysis**					
TLR 2 (% +ve)	29	30	100 ± 0	100 ± 0	0.9722
TLR2 MedFI	29	30	289 ± 18	219 ± 12	0.0062
TLR4 (% +ve)	30	30	52 ± 3	48 ± 3	0.2351
TLR4 MedFI	30	30	9 ± 1	8 ± 0	0.4383
CD86 (% +ve)	29	30	99 ± 0.3	100 ± 0.1	0.9004
CD86 MedFI	29	30	80 ± 4	71 ± 4	0.0893
**Serum cytokines**					
IL-1Rα	31	31	343 ± 75	203 ± 43	0.0127
IL-6	31	31	101 ± 49	44 ± 16	0.0291
IL-8	31	31	40 ± 6	28 ± 2	0.0564
IL-10	30	31	57 ± 16	127 ± 73	0.0268
G-CSF	31	31	81 ± 11	83 ± 21	0.3788
TNF-α	31	31	179 ± 94	113 ± 41	0.7353
MCP-1	31	31	247 ± 118	99 ± 29	0.1573

G-CSF, granulocyte-colony stimulating factor; IL, interleukin; MCP-1; monocyte chemoattractant protein-1; MedFI, median fluorescence intensity; MODS, multiple organ dysfunction syndrome; TLR, Toll-like receptor; TNF-α, tumour necrosis factor-alpha.

Patients who developed MODS had a significantly higher ISS and a lower base excess at admission (Table 1). Thus, binary logistic regression analysis was performed to account for any potential effects of injury severity and haemorrhagic shock on the relationship we had identified between NKT and CD3^+^8^+^ number and the development of MODS. Our analysis found that only NKT cell counts exhibited an independent association with MODS after adjusting for ISS and base excess (Table 9). To examine this output in further detail, we explored the effect that increasing the value of NKT cell number by quartiles had on developing MODS. As shown in Table 10, we found that increasing the value of NKT cell number from its first quartile (84.5 x 10^6^/L) to the second quartile (185.5 x 10^6^/L) corresponded to an OR of developing MODS of 1.63 (95% CI 1.081–2.463), whilst increasing the value of NKT number from its second quartile (185.5 x 10^6^/L) to the third quartile (416.8 x 10^6^/L) corresponded to an OR of developing MODS of 3.06 (95% CI 1.195–7.880).

**Table 9:** Binary logistic regression analysis examining the association between NKT and CD3^+^8^+^ cell number and the development of MODS.

Variable	Β (SE)	Odds ratio (95% CI)	*p*-Value	C statistic^1^
**NKT cell number**				
Intercept	−4.986 (1.355)	0.007 (0.000–0.100)	0.0002	0.892
Base excess	−0.200 (0.128)	0.819 (0.637–1.052)	0.120	
ISS	0.115 (0.038)	1.122 (1.041–1.209)	0.003	
NKT cell number	0.005 (0.002)	1.005 (1.001–1.009)	0.020	
**CD3**^**+**^**8**^**+**^ **cell number**				
Intercept	−4.592 (1.235)	0.010 (0.001–0.114)	0.0002	0.876
Base excess	−0.214 (0.109)	0.807 (0.652–1.000)	0.049	
ISS	0.113 (0.037)	1.120 (1.041–1.204)	0.002	
CD3^+^8^+^ cell number	0.0009 (0.0006)	1.001 (0.999–1.003)	0.169	

^1^Corrected for overfitting using internal validation with 9,999 bootstrap resamples.

NKT cell number: R^2^ value of 0.625, le Cessie-van Houwelingen goodness-of-fit test *p*-value of 0.409, Brier score of 0.123. MODS *n* = 24, No MODS *n* = 28. CD3^+^8^+^ cell number: R^2^ value of 0.563, le Cessie-van Houwelingen goodness-of-fit test p value of 0.409, Brier score of 0.135. MODS *n* = 27, No MODS *n* = 29. CI, confidence interval; ISS, injury severity score; MODS, multiple organ dysfunction syndrome; NKT, natural killer T.

**Table 10:** Illustrative odds ratios for development of MODS based on changes in values of NKT number.

Variable		Change in NKT cell number	Odds ratio (95% CI)
NKT cell number			
From	To		
84.5	185.5	101	1.632 (1.081–2.463)
185.5	416.8	231.3	3.069 (1.195–7.880)
84.5	416.8	332.3	5.007 (1.292–19.408)

Output based on the NKT model summarised in Table 9. Analysis based on MODS *n* = 24 and no MODS *n* = 28. CI, confidence interval; MODS, multiple organ dysfunction syndrome; NKT, natural killer T.

## Discussion

Here, via the analysis of 89 blood samples obtained from patients within 1 hour of injury, the so-called “Golden Hour”, we have provided for the first time, to our knowledge, a detailed description of the ultra-early changes that occur in the composition, function, and/or phenotype of the innate and adaptive arms of the immune system post-trauma. We have shown that accompanying the well-documented “cytokine storm” of raised pro- and anti-inflammatory cytokines was a complex immune response that encompassed features of both immune activation and suppression. For example, in the background of leukocytosis, ex vivo phenotyping revealed increased and decreased surface expression of CD11b and CXCR2, respectively, on neutrophils and increased surface TLR2, TLR4, and CD86 expression on monocytes, whilst the presence of nuclear DNA and CitH3 in plasma samples suggested the rapid induction of neutrophil function in the form of NET generation. These signs of immediate immune activation occurred alongside alterations consistent with immuneparesis, such as elevated numbers of suppressive CD16^BRIGHT^ CD62L^DIM^ neutrophils and CD14^+^HLA-DR^low/−^ monocytes, a lack of responsiveness of neutrophils to fMLF stimulation, and impaired leukocyte cytokine production following LPS stimulation. Thus, together with the results of previous genomic and functional studies [10,18], our data challenge the traditional SIRS:CARS concept in which a sequential immune and inflammatory response to injury is suggested and in which the CARS response is proposed to follow the SIRS response in order to restore homeostasis [38]. Rather, the increasing consensus is that the SIRS and CARS responses are largely concomitant features of the postinjury immune response, and our study reveals that these are evident within minutes of trauma.

Analysis of neutrophil function across our 3 postinjury time points revealed a significant reduction in PMA-induced ROS production and a reduced responsiveness to bacterial peptide stimulation, with the latter defect demonstrated by reduced shedding of CD62L following fMLF treatment. These aberrations were accompanied by significantly elevated numbers and frequencies of both highly mature CD16^BRIGHT^ CD62L^DIM^ neutrophils and IGs, with the emergence of IGs into the circulation borne most likely from a stress-induced early mobilisation of immature precursors from the bone marrow. The increase in mature neutrophil numbers is most likely a result of glucocorticoid-induced demargination [39], as up to 50% of neutrophils are marginated in the endovascular lining in the noninjured state and can be very rapidly mobilised into the circulation to produce the neutrophilia of trauma. Compared to their CD16^BRIGHT^ CD62L^BRIGHT^ counterparts, CD16^BRIGHT^ CD62L^DIM^ neutrophils exhibit reduced ROS production, a trait shared by IGs, who also show reduced responsiveness to fMLF challenge [40–42]. Thus, the heterogeneous neutrophil pool consisting of highly mature subsets and IGs that developed postinjury could be 1 factor underlying the altered neutrophil function we describe here. In addition to reduced ROS generation and responsiveness to fMLF, immature neutrophils exhibit impaired phagocytosis, chemotaxis, and bacterial killing [40] aberrations, which in the case of phagocytosis and chemotaxis, mirror the ex vivo behaviours described for neutrophils isolated from trauma patients [3–5]. Thus, the release of IGs into the circulation post-trauma may impact upon many facets of neutrophil antimicrobial defence, and given that IG numbers peaked in samples acquired in the immediate aftermath of injury, defects in neutrophil function may be apparent within minutes of trauma.

At all 3 postinjury time points, trauma patients presented with significantly elevated numbers and/or frequencies of suppressive CD14^+^HLA-DR^low/−^ monocytes and CD16^BRIGHT^ CD62L^DIM^ neutrophils. A rich source of IL-10 and arginase [43,44], CD14^+^ HLA-DR^-/low^ monocytes have been shown in vitro to inhibit T cell activation, suppress NK cell cytotoxicity, and promote the expansion of T regulatory cells [45–47], whilst via their generation of ROS, CD16^BRIGHT^ CD62L^DIM^ neutrophils have been reported to suppress T cell proliferation [34]. Thus, as proposed for other conditions in which systemic immune suppression has been described [43], the emergence into circulation of immune modulatory subsets could offer 1 potential mechanistic explanation for the development of immuneparesis after injury. On this theme of post-trauma immuneparesis, data are beginning to emerge that suggest that in addition to triggering cell activation, prior exposure to DAMPs can induce a state of functional tolerance in immune cells. In monocytes, it has been shown that preconditioning with heat shock protein-70, a nuclear-derived DAMP whose plasma concentrations are significantly elevated within minutes of injury [10], culminates in impaired cytokine production upon secondary LPS challenge [48], whilst for neutrophils, prior exposure to fMLF, which activates the same signalling pathways as mitochondrial-derived N-formyl peptides, results in aberrant responses in subsequent functional assays [5]. Thus, in line with previous studies [5,10], we speculate that via immune cell activation, an immediate trauma-induced elevation in circulating DAMPS may induce a state of functional tolerance that culminates in impaired functional responses in vitro.

At the 4–12- and 48–72-hour postinjury time points, we detected a significant reduction in PMA-induced neutrophil ROS production, an impairment that has previously been described for neutrophils isolated from patients following thermal [49] and isolated TBI [50]. In the case of thermal injury, the reduction in ROS production was attributed to a deficiency in p47-phox and p67-phox [49], 2 cytosolic components of NADPH oxidase, a multi-subunit enzyme that initiates the respiratory burst. In the only human-based study to our knowledge that has examined NADPH oxidase expression in immune cells post-trauma, Liao and colleagues reported elevated expression of the membrane-residing subunit gp91-phox in leukocyte homogenates from TBI patients [4]. However, the group did not investigate the expression of other membrane or cytosolic subunits of NADPH oxidase, nor did they study neutrophils in isolation [4]. Thus, it is currently unclear as to whether, as reported for burns patients [49], reduced expression of NADPH oxidase subunits could underlie the impairment in PMA-induced ROS generation we found postinjury. A critical step in the activation of NADPH oxidase is the phosphorylation of its subunits. A pharmacological mimetic of diacylglycerol, PMA works in conjunction with intracellular calcium to activate the classical isoforms of the serine/threonine protein kinase, protein kinase C (PKC), whose substrates include the NADPH oxidase subunits gp91-phox, p47-phox, and p67-phox [51–53]. Interestingly, blunted intracellular calcium mobilisation following inflammatory agonist stimulation has been reported for neutrophils isolated from major trauma patients [54]. Thus, an additional/alternative explanation for the impaired ROS production in response to PMA postinjury could be reduced PKC activation due to a lack of intracellular calcium.

Recently, in the setting of critical illness, Hirose et al. [55] failed to detect CitH3, a protein that decorates the DNA backbone of NETs [33], in blood samples obtained from trauma patients at the time of their admission to an ICU. The authors proposed that its absence reflected the fact that samples had been acquired too soon following the onset of critical illness for NET formation to have occurred [55]. However, our preliminary data showing the presence of CitH3 in plasma samples collected from patients within minutes of trauma suggest their samples were obtained too late (rather than too early), since NET production appears to be a feature unique to the ultra-early immune response to trauma. Our observation of immediate NET formation raises the question as to how a feature of neutrophil antimicrobial defence involved in pathogen entrapment and neutralisation can be triggered so quickly following sterile injury. Recently, it was reported that neutrophils treated in vitro with the nuclear-derived DAMP high-mobility group box 1 (HMGB1) generate NETs within 1 hour of stimulation [56], and elevated plasma levels of this DAMP have been recorded within 30 minutes of major injury [57]. Thus, an immediate release of HMGB1 from damaged tissue may be 1 mechanism underlying the rapid formation of NETs post-trauma.

Our study highlights how differences in sample timing can influence our understanding of the postinjury immune response. For instance, elevated absolute numbers of B, NK, NKT, CD3^+^, CD3^+^4^+^, and CD3^+^8^+^ cells were recorded in blood samples acquired from patients within minutes of injury, thereby revealing an immediate post-trauma leukocytosis. However, at the 4–12-hour time point, patients presented in a state of lymphopenia, with significantly reduced numbers of circulating CD56^BRIGHT^ NK cells as well as CD3^+^ and CD3^+^4^+^ T cells. Similarly, we found features of the ultra-early immune response, such as the presence of plasma-residing CitH3 and increased surface densities of CD16 on neutrophils and TLR2, TLR4, and CD86 on monocytes, were absent from samples acquired from patients 4–12 hours postinjury. Conversely, only in blood samples obtained in the hours following injury were defects in neutrophil ROS generation upon PMA stimulation and reduced HLA-DR expression by monocytes observed, thereby demonstrating that not all aspects of trauma-induced changes in immunity occur within minutes of injury.

Manson et al. recently reported that lymphocyte activation within 2 hours of injury was associated with later development of MODS [9]. Based on this observation, we performed exploratory analysis of our dataset to investigate whether any features of the ultra-early immune response to injury were related to patient outcome. After controlling for a false discovery rate of 5%, we identified a potential association between elevated absolute numbers of NKT and CD3^+^8^+^ cells within minutes of injury and the subsequent development of MODS. However, after adjusting for ISS and base excess, only NKT cell counts exhibited an independent association with MODS. It must be stressed that because of our small patient cohort and the fact that our study was not designed from the outset to test this relationship directly, this association may be limited to our dataset, and thus, we cannot generalise our data to all trauma patients from whom blood samples could be acquired within 1 hour of injury. Thus, rather than a conclusive finding, our observation has generated a hypothesis that future adequately powered prospective cohort studies should investigate in order to establish whether the immune response elicited within minutes of trauma is indeed associated with poor clinical outcome.

The relatively high level of MODS for this study cohort may at first seem unusual since inclusion criteria for the study was ISS > 8. However, the mean ISS for the study cohort was 24, representing a relatively high injury burden. The statistically significant difference between the ISS for those who developed organ failure (mean ISS 31) versus those who did not (mean ISS 18) is in keeping with what might be expected in the trauma population. It is also important to note that none of our patients received blood products in the prehospital setting, thereby ruling out the possibility that the immediate dysfunction we observed was a consequence of the immune modulatory effects of blood transfusion [58,59].

The major limitation of our study is its relatively small sample size and the fact that it was conducted at a single major trauma centre, meaning the results generated require validation in larger independent cohorts. That said, given the logistical difficulties associated with obtaining prehospital samples, our study has provided novel data that should serve to stimulate future prehospital-based research to answer questions we did not address here. For example, although we performed phenotypic analysis, we did not assess T or B cell function, nor did we examine the phagocytic capacity of innate immune cells. Moreover, we did not examine any aspect of immune cell signalling, which may have provided a mechanistic insight into the immune changes we observed. A previous study reported that reduced immune function 48–96 hours postinjury was accompanied by impaired intracellular signalling [17], and our finding of reduced PMA-induced ROS production by neutrophils postinjury points to a potential impairment in signalling pathways distal to the plasma membrane. In addition, our study recruited a very heterogeneous patient group that included subjects with and without head injuries, though we excluded those with isolated head injuries in this study. Interestingly, a higher incidence of head injury was observed amongst patients who developed MODS, and thus, future studies enrolling a larger number of patients should consider patients with extracranial injuries in the presence or absence of head injury as separate groups. Finally, our study design was at risk of selection bias, since only patients that underwent venepuncture during prehospital evacuation could be enrolled. This is a procedure that takes a few minutes and may have only been performed at certain times or during prehospital treatment that would allow such an addition to standard care. This may have inadvertently increased the risk of selection bias towards less severely injured patients, although maintenance of a screening log helped reduce this risk.

In summary, our study has highlighted the dynamic nature of the immune response to trauma and shown at the functional and phenotypic level that immune alterations consistent with activation and suppression are evident within 1 hour of injury, thus supporting the idea of an immediate and concomitant induction of the SIRS and CARS responses post-trauma.

## Supporting information

S1 Data Database of the study’s raw data. (XLSX)

S1 Fig Serum concentrations of monocyte chemoattractant protein-1 (MCP-1) post-trauma. The number of patient and healthy control (HC) samples analysed is indicated below each time point. The horizontal line for HC data depicts the median value.
(TIF)

S2 Fig Flow diagram showing immune and inflammatory analysis of blood samples obtained within 1 hour of traumatic injury from patients who subsequently did or did not develop MODS. Insufficient sample volume and equipment breakdown accounts for the differences in patient numbers between each parameter analysed. LOS, length of stay; MODS, multiple organ dysfunction syndrome; NK, natural killer; TLR, Toll-like receptor.
(TIF)

S1 Table Demographics of healthy controls (HCs). (DOCX)

S1 Text Study protocol. (DOC)

S2 Text Strengthening the Reporting of Observational Studies in Epidemiology (STROBE) checklist. (DOC)

## Abbreviations

A/P: assault/penetrating
AIS: abbreviated injury scale
CARS: compensatory anti-inflammatory response syndrome
CF-DNA: cell-free DNA
CI: confidence interval
CitH3: citrullinated histone H3
DAMP: damage-associated molecular pattern
fMLF: formyl-methionine-leucine-phenylalanine
GCS: Glasgow coma scale
G-CSF: granulocyte-colony stimulating factor
HC: healthy control
HLA-DR: human leukocyte antigen DR
HMGB1: high-mobility group box 1
ICU: intensive care unit
IG: immature granulocyte
IL: interleukin
IL-1Ra: interleukin-1 receptor antagonist
ISS: injury severity score
LOS: length of stay
LPS: lipopolysaccharide
MCP-1: monocyte chemoattractant protein-1
MedFI: median fluorescence intensity
MFI: mean fluorescence intensity
MODS: multiple organ dysfunction syndrome
mtDNA: mitochondrial DNA
nDNA: nuclear DNA
NET: neutrophil extracellular trap
NHS: National Health Service
NIHR SRMRC: National Institute for Health Research Surgical Reconstruction Microbiology Research Centre
NISS: new injury severity score
NK: natural killer
NKT: natural killer T
NS: nonsignificant
OR: odds ratio
PKC: protein kinase C
PMA: phorbol 12-myristate 13-acetate
ROS: reactive oxygen species
RTC: road traffic collision
SIRS: systemic inflammatory response syndrome
STROBE: Strengthening the Reporting of Observational Studies in Epidemiology
TBI: traumatic brain injury
TEMRA: terminally differentiated effector memory
TNF-α: tumour necrosis factor-alpha
TOI: time of injury
WBC: white blood cell
