# Supplementary figures and images for: Prehospital immune responses and development of multiple organ dysfunction syndrome following traumatic injury: A prospective cohort study
Source: PLoS Med. 2017 Jul 18;14(7):e1002338. doi: 10.1371/journal.pmed.1002338 (PMC5515405; doi:10.1371/journal.pmed.1002338)

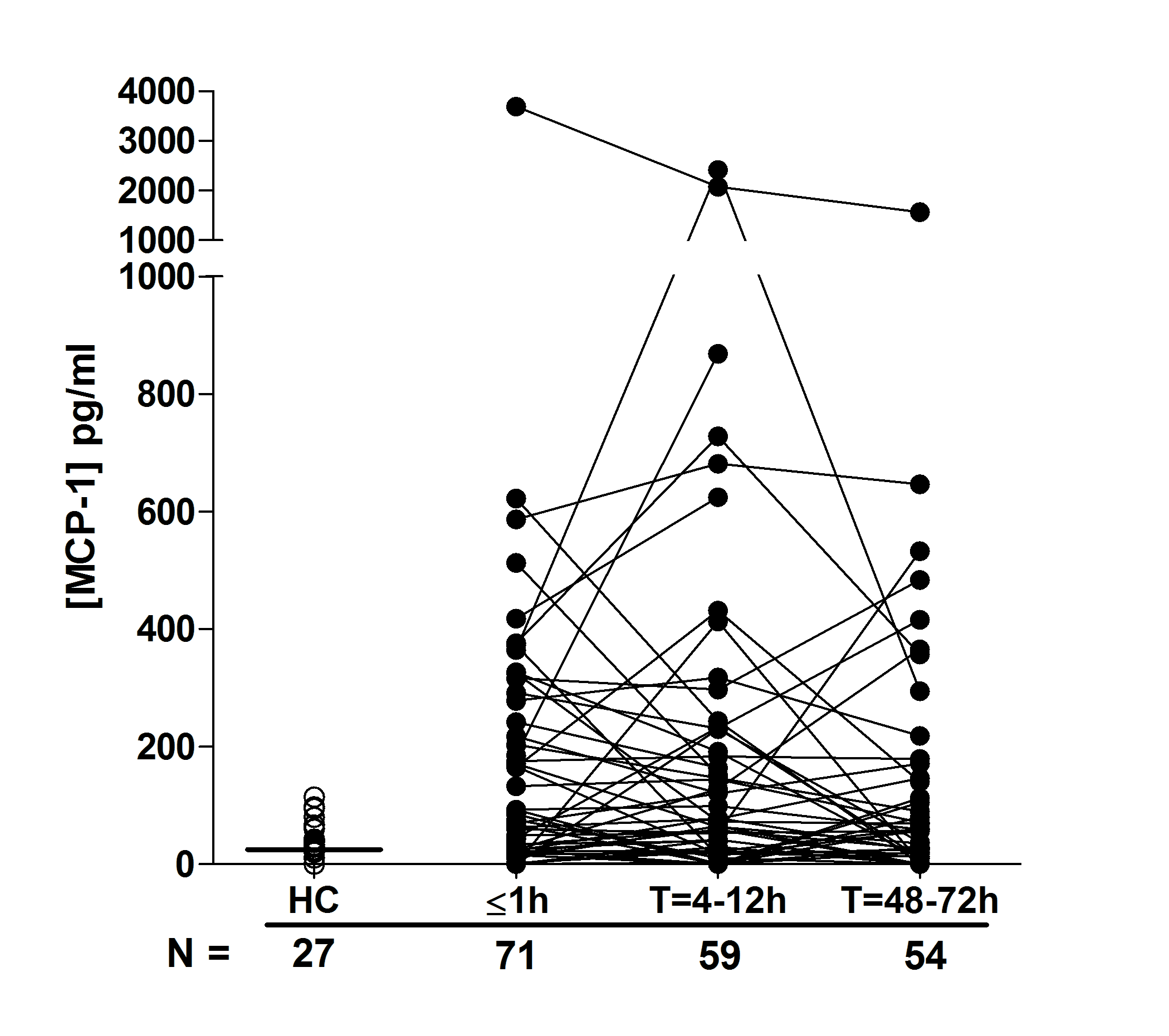

Supplement: S1 Fig — The number of patient and healthy control (HC) samples analysed is indicated below each time point. The horizontal line for HC data depicts the median value. (TIF) [file pmed.1002338.s002.tif]

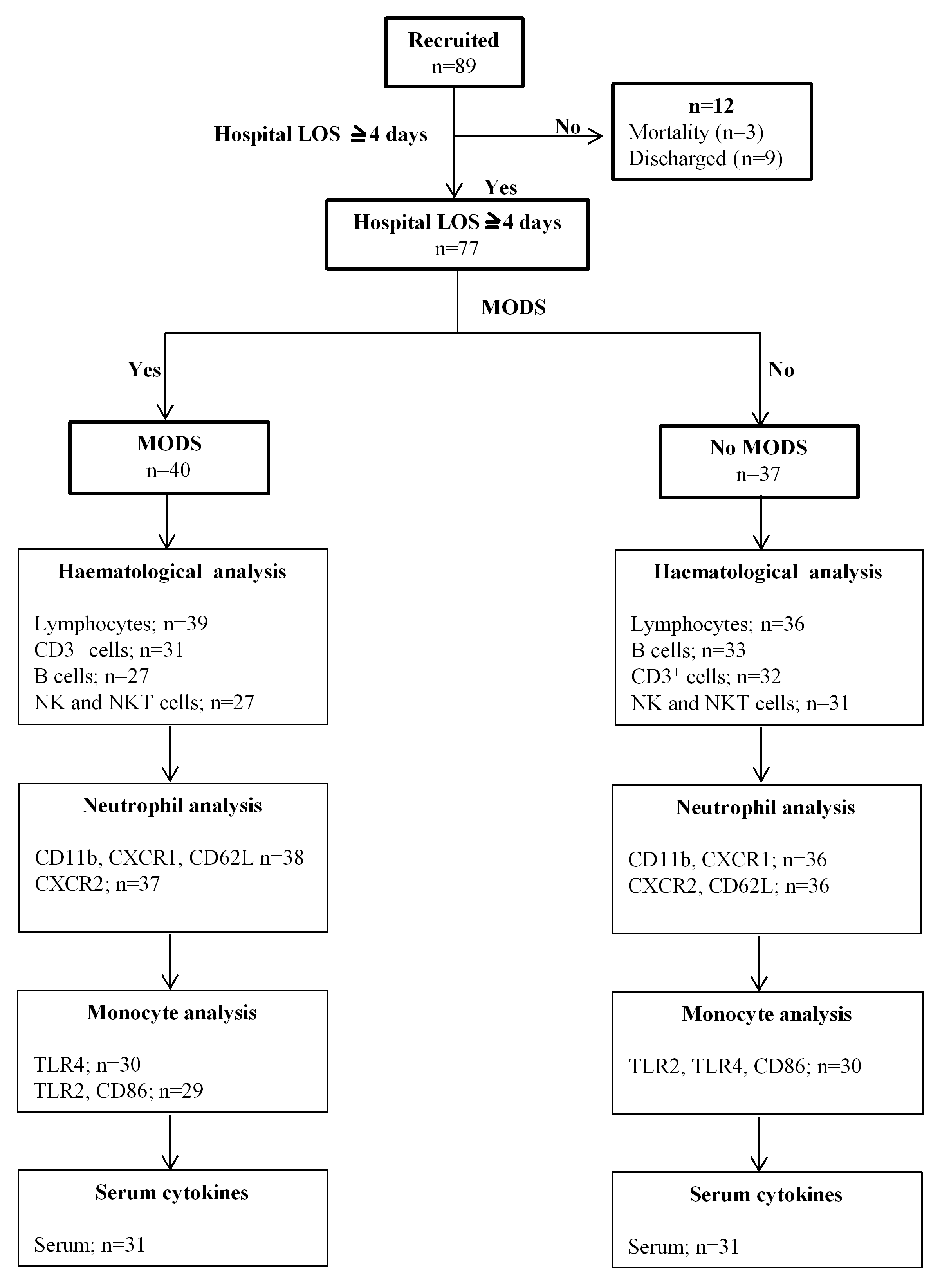

Supplement: S2 Fig — Insufficient sample volume and equipment breakdown accounts for the differences in patient numbers between each parameter analysed. LOS, length of stay; MODS, multiple organ dysfunction syndrome; NK, natural killer; TLR, Toll-like receptor. (TIF) [file pmed.1002338.s003.tif]
