# Supplementary material for: Prehospital immune responses and development of multiple organ dysfunction syndrome following traumatic injury: A prospective cohort study
Source: PLoS Med. 2017 Jul 18;14(7):e1002338. doi: 10.1371/journal.pmed.1002338 (PMC5515405; doi:10.1371/journal.pmed.1002338)
Supplement: S1 Table — (DOCX) [file pmed.1002338.s004.docx]

|  | **Patients**  **(n=89)** | **Healthy Controls**  **(n=116)** | **p Value** |
| --- | --- | --- | --- |
| **Age, years** | 41 (18-90) | 38 (19-82) | 0.372 |
| **Gender (M:F)** | 75:14 | 87:29 | 0.106 |

Data are expressed as mean (range) unless indicated otherwise.
