# Supplementary material for: Prehospital immune responses and development of multiple organ dysfunction syndrome following traumatic injury: A prospective cohort study
Source: PLoS Med. 2017 Jul 18;14(7):e1002338. doi: 10.1371/journal.pmed.1002338 (PMC5515405; doi:10.1371/journal.pmed.1002338)
Supplement: S1 Text — (DOC) [file pmed.1002338.s005.doc]

**Brain Biomarkers After Trauma Study (BBATS)**

**Protocol V1.1 dated 27th December 2013**

**REC ref** *13/WA/0399*

The study is a collaboration between The University of Birmingham, University Hospitals Birmingham NHS Trust (UHB) and Defence Scientific and Technology Laboratories (DSTL).

**Sponsor: University of Birmingham**

**Principle Investigator:**

**Mr Antonio Belli MD, FRCS, FRCS (SN)**

School of Clinical and Experimental Medicine
College of Medical and Dental Sciences
Institute of Biomedical Research (West), Room WX 2.61
University of Birmingham
Edgbaston
Birmingham
B15 2TT

Tel: 0121 414 4497 Email: a.belli@bham.ac.uk

**Researchers**

**Mr David Davis Neurosurgical Registrar**

**Mrs Aisling Clarkson Trauma Research Sister**

**Mr Zhangzie Su Clinical Research Fellow**

**Maj James Mitchell Medical Officer RCDM**

**Research sites**

1. Participants:
   1. Will have been admitted to UHB
   2. Sampling will take place at UHB wards or OPD
2. Measurements. Analysis of samples will be conducted in:
   1. Routine pathology laboratories of UHB
   2. University of Birmingham Research Laboratories, IBR
   3. DSTL.

**Background**

Through the collaboration of the UHB, DSTL and University of Birmingham we wish to characterise the biomarker response to traumatic brain injury and define the roles of biomarkers relating to injury detection, diagnosis, monitoring, classification, prognosis and treatment. This will form a basis for future interventional studies to improve outcome from traumatic brain injury.

This study draws from two previous studies, Biomarkers of Brain Injury and Steroids and Immunity from Injury through to Rehabilitation study. The former aimed to identify novel biomarkers in a severe brain trauma population; the latter included a sub-population of severe brain trauma and looked at a small range of biomarkers. The major weaknesses in both of these studies were a limited appraisal of outcome following brain trauma as opposed to severity at inclusion and limited follow-up of participants. We aim to build on these studies with a broader population and comprehensive assessment of outcome.

Studies carried out have not fully defined the role of biomarkers in clinical practice; few have made a transition to routine practice.

*Traumatic Brain Injury (TBI)*

There is increasing interest in TBI and it remains a leading cause of morbidity and mortality in particular affecting the young with a male preponderance. Although previously reported to be an untreatable cause of mortality there have been improvements, particularly when treatment is given in specialist neurosurgical centers irrespective of need for surgical intervention as recommended by NICE in the UK. At present treatment is largely supportive and investigational techniques have not changed apace clinically with the research field.

*Biomarkers in Brain Injury*

A biomarker in the broadest sense is any characteristic that may be objectively assessed to provide an indicator of a normal or abnormal disease or physiological state of an organism, this includes imaging and laboratory tests along with more simple clinical findings. Biomarkers are used in other clinical settings such as Troponin for cardiac damage.

There has been much interest in the development of biomarkers for neuro-trauma though this has yet to lead to a change in clinical practice. Previous research has assessed potential biomarkers, for instance Creatine kinase, glial fibrillary acidic protein, myelin basic protein and S100B. S100B has been the most intensely studied and has since been shown not to be specific to CNS damage.

CT remains the mainstay imaging technique in the acute phase of assessment for brain injury though has a limited ability to demonstrate pathology, MRI has increased sensitivity for some aspects of trauma but both are outstripped by a host of new techniques. Diffusion Tensor Imaging (DTI) is a development of MRI relying on standard equipment with different processing techniques. It has much improved sensitivity for axonal injury, the hallmark of TBI. Regarding laboratory markers, these are substances released generally in response to or as an effect of injury in this context and may be assayed in order to provide an insight into many factors surrounding an injury, such as severity.

This study aims to improve on previous clinical studies by investigating a range of potential biomarkers in serum, diasylate, CSF and imaging in brain injured and non-brain injured (control) trauma patients with comprehensive outcome measures. The benefits to patients enrolled in this study are minimal.

In the long term we hope these biomarkers might improve the diagnosis, monitoring and prognostication of brain trauma thus allowing better targeted treatment and potentially revealing new avenues of treatment.

**Aim**

To characterise the relationship between biomarkers, brain injury severity and outcome.

**Hypotheses**

The hypothesis to be tested is that biomarkers of neurotrauma may give specific prognostic information for an individual patient to inform acute and sub-acute treatment stratification and future prognosis. Measurements of biomarkers taken after trauma will be correlated with outcome measures at 6 and 12 months.

Secondary hypotheses are that novel biomarkers exist and may be identified; genetic factors are important modulators of outcome.

**Clinical Setting**

University Hospital Birmingham is a fast developing major trauma centre and tertiary referral neurosurgical centre seeing a large number of both TBI and general trauma casualties from across the West Midlands. The latter group providing non-head injury comparison. Analysis for biomarkers will be performed at the University of Birmingham and DSTL.

**Study Design**

This is a cohort study to categorise the traumatic brain injury population at UHB. Brain biomarkers, serum, micro-diasylate, CSF and imaging will be measured in two groups of patients. Outcome will be assessed by TMS threshold, ERP, ERG, VEP and cognitive and psychological questionnaires will be performed. The two groups of patients will be:

The study group - patients who have suffered a head injury presenting or transferred to UHB.

The control group - patients admitted with non-head trauma matched to the study group by ISS severity and demographic.

Patients for inclusion will be identified at point of injury or as soon as possible after presentation to UHB upto 1 hour post injury. For samples taken pre-hospital where patients are unable to consent these will be taken, subject to a favourable REC opinion under the Mental Capacity Act ‘waiver’ for research in emergency situations. Advice will be sought from a consultee as soon as practicable following presentation to hospital and consent will be sought from the patient once capacity has been regained. Information in the form of the included Patient Information Sheets or Consultee Information Sheet will be provided; consent or advice will be recorded on the appropriate written forms annexes c-i.

*Inclusion criteria:*

Study group - Patients who have sustained a head injury resulting in presentation to UHB within 1 hour of injury.

Control group - non-head trauma patients matched to control by severity (ISS) and demographic.

*Exclusion criteria:*

Children under the age of 18 years. For the control group those with a recent head injury or known neurological condition.

Our conservative assumption is that 80% of head injured patients will show a positive biomarker response with 10% of non-head injured patients showing a positive response. Using fishers Exact test (power0.9; alpha 0.05; 1:1 control subject ratio) would predict 10 patients in each group. However head injuries and trauma in general demonstrates much heterogeneity and to allow comparison across modalities and sub-groups we intend to recruit up-to 100 patients to the study group and the control group.

*Sampling - Blood*

Sampling of blood will be performed at regular intervals beginning at point of injury through to follow-up at six months post injury. During emergency treatment at point of injury venous access is gained to allow the delivery of drugs and fluids. Blood samples are not usually withdrawn as there are no current clinical investigations available at that time-point. We propose to withdraw 20mls of venous blood for early biomarker analysis. Blood samples are taken routinely over the following days of admission to monitor various factors, where feasible research samples will be withdrawn alongside clinical samples.

Blood samples will be analysed for an array of biomarkers with particular interest in early markers with short half-lives. Specifically S100B, Glial fibrillary acidic protein, myelin basic protein, micro-particle analysis and metabolomics assays will be performed. Genetic analysis will be performed initially looking at susceptibility or protective factors with differing mtDNA haplotypes.

*Micro-dialysis*

Intra-cranial pressure monitoring and more lately micro-dialysis catheter monitoring are part of routine clinical care for head trauma allowing monitoring of intra-cranial pressure and brain oxygen tension; important clinical variables. Micro-dialysis is a technique that involves the placement of a catheter containing a semi-permeable membrane into a tissue. A fluid is then circulated over this membrane and small-molecule substances in the tissue will then diffuse across the membrane allowing analysis to be performed to detect those of interest, this fluid is known as a diasylate. Although this technique was first developed 50 years ago it has only recently been adopted into routine clinical practice in certain centers. Presently the diasylate is discarded following bedside measurement of oxygen, however this fluid could provide a wealth of potential biomarkers, we propose to recover the fluid for analysis. Control patients would not be expected to have ICP monitors of micro-dialysis catheters as these are solely used in the setting of neurosurgical problems.

*CSF*

CSF (cerebrospinal fluid) samples may be withdrawn as part of routine clinical management, particularly where extra-ventricular drains or similar have been placed. Where this happens we propose to withdraw an additional sample of CSF for analysis.

*Imaging*

Imaging will be performed on a random selection of patients as detailed below. MRI, MRS, and DTI sequences will be attained. The initial scans will be interpreted using the imaging findings as biomarkers for trauma. Specifically structural and micro-haemorrhages on MRI, N-acetylaspartate (NAA), choline, creatinine, myoinositol with Magnetic Resonance Specroscopy and changes in fractional anisotropy with Diffusion Tensor Imaging as a marker of axonal disruption.

*Outcome Measures*

A marked weakness in previous studies has been the lack of comprehensive outcome assessment as opposed to assessment of injury severity shortly after injury. Outcome following injuries can be very heterogeneous and prognostication is often inaccurate. For this reason all-round assessment of outcome should allow better extrapolation of the clinical usefulness of potential biomarkers.

At follow-up participants will complete a number of psychological and cognitive questionnaires to categorise various aspects of outcome following their injury. Participants will also undergo measurement of TMS motor thresholds before and after a motor learning exercise. EEG will be performed to measure p300 and VEP, ERG will be recorded.

*Cognitive and psychological questionnaires*

The complexity of the brain leads to a wide array of effects following injury. A plethora of tests have been developed to try to capture these and relate to severity of injury. We have selected short questionnaire based tests to focus on the characteristic sequelae of neuro-trauma, cognitive effects including memory, post-traumatic stress symptoms, depression and anxiety. The Test of Memory Malingering TOMM and Neurological assessment battery NAB will be used and will augment those used in routine clinical practice. Outcome will be assessed by scores against published validation populations. High scores indicating abnormality in the domain tested specific to the individual test. TOMM provides validity to other administered tests and is not an outcome measure of itself.

*Neurophysiology*

Many techniques have been developed for and applied to the study of brain disorders and the impact on brain function. A number of neurophysiological techniques may be used to investigate specific pathways or attributes. Trans-cranial magnetic stimulation involves the use of magnetic fields to stimulate specific groups of neurons, the effect of that stimulus may then be measured, for instance in the motor cortex by electro-myogram (EMG) recording of the muscle that area represents. Responses of neurons to the stimulus can be affected by learning activities; the affect of which may be altered following brain injury. Other techniques may be used to study other brain pathways, event related potentials are generalised electrical responses of the brain to a stimulus. p300 is one such potential that may be measured by EEG recording of a subject whilst undertaking a task; the oddball paradigm is an auditory task where-by an unexpected auditory stimulus is introduced allowing the measurement of the p300 potential it triggers. These responses depend on communication between diffuse brain areas and study of them provides insights into that communication in health and disease. These principles have also been applied to the visual system, an electro-retinogram (ERG) may record the retinal response to visual stimuli and a limited version of EEG may record the response of the occipital visual centers to similar stimuli, both can provide important information regarding relevant brain areas following injury.

Outcome will be assessed by results outside of published norms. TMS measurements will look for a loss of the threshold change expected after a learning activity in those suffering the sequelae of head trauma. Outcome for event related potentials will be a delay or absence of the potential.

**Methodology**

1. Patient Recruitment: All trauma patients with a survivable injury and ISS (decision) will be entered into the study. 100 trauma patients with a brain injury and 100 trauma patients without a brain injury will be studied. The study team will liaise with the emergency department at UHB for local trauma.
   1. Patient journey: Demographics will be entered into a patient database, a schedule of sampling will then be generated to allow the patient to be followed through the study.
   2. Insults: initial injury and insults - baseline GCS score, post-traumatic amnesia, surgical intervention, infection, sepsis, ICP, cerebral blood flow and oxygenation, seizure.
   3. Timings: Admission, surgery, ventilation days, ICU days, discharge, rehabilitation, time to recovery.
2. Measurements and sample collection: The following measurements will be made on samples taken from point of injury through to six month follow-up. The schedule appears in table 1.
   - 1. *Venous blood*. Venous access for drugs and fluids is gained early in trauma care at point of injury, however blood would not routinely be withdrawn as there are no useful clinical tests at this stage currently. We propose to withdraw 20mls at this point for analysis of the early biomarker milieu. An additional 20mls of venous blood will be taken at subsequent time-points. These will be analysed for biomarker levels and metabolomics. Genomic analyses will be carried out on mitochondrial and nuclear DNA, including mitochondrial DNA haplogroups, Apoliprotein E haplotypes and genes of the MToR pathway.
   1. *Micro-diasylate*. Micro-dialysis is used in routine clinical practice to monitor cerebral perfusion. The small amount of diasylate generated would normally be discarded in clinical practice, we propose to recover it for analysis. The diasylate will be snap frozen following recovery and sent to DSTL for analysis.
   2. *CSF*. Where CSF is withdrawn in normal clinical practice an additional 5 mls will be withdrawn for biomarker analysis.
   3. *Imaging.* MRI, MRS, DTI, will be performed in accordance with the schedule at table 2.
      1. MRI: T1, T2, FLAIR, DWI, ADC sequences will be acquired on a 1.5T scanner in the UHB inpatient imaging department.
      2. MRS: sequences will be acquired on a 1.5T scanner in the UHB inpatient imaging department.
      3. DTI: sequences will be acquired on a 1.5T scanner in the UHB inpatient imaging department.
   4. *Test Of Memory Malingering (TOMM).* The TOMM is administered by a trained clinical psychologist, it provides a quantification of effort and helps to validate findings of routine clinical questionnaires, it is not transparent to the participant. TOMM score sheet annex l. *Neurological Assessment Battery (NAB)*. A short version is administered which quantifies cognitive sequelae, it is in questionnaire format.
   5. *Trans-cranial Magnetic Stimulation (TMS).* This will be used to explore the neurophysiology behind how the injured brain learns using protocols that are well established and validated in healthy volunteers. Participants will undergo two bouts of single pulse TMS at frequencies no greater than 0.67 Hz before and after a period of learning. The learning task involves seating the participant in front of a computer and tasking them to track a cursor presented on the screen as accurately as possible. For each of the target lines presented, performance will be quantified as root mean square error between the actual and target traces. The change in activation threshold will be measured before and after which provides insight into the underlying changes. This will be performed at 1 year.
   6. *EEG.* The p300 event related potential will be recorded for participants using an auditory oddball paradigm. Latency will be recorded at the scalp electrode with greatest response, EEG itself is as per standard neurophysiological clinical practice. This will be performed at 1 year.
   7. *ERG*. This is recorded using a standard ISCEV protocol for flash and pattern and multifocal stimuli. Participants have three electrodes placed on the forehead and either side of the eyes and a very fine DTL loop electrode placed in the inferior conjunctival fornix which is painless. Recordings of responses to the stimuli are taken following light and dark adaptation. This will be performed at 1 year.
   8. *VEP*. A limited form of EEG is recorded over the occipital cortex to record higher level responses to flash and pattern and multi-focal stimuli, again to the ISCEV standard protocols. This will be performed at 1 year.
   9. *Pupillometry*. This utilises a device to accurately and sensitively assess the pupillary reflexes giving information on the pathways subserved. This will be performed during the first 30 days.
3. Locations:
   1. Sample collection at point of injury will be conducted by West Midlands Ambulance Service.
   2. Routine blood analysis will be performed as per normal clinical practice by UHB haematology and biochemistry laboratories.
   3. Biomarker assays will be performed at DSTL and UoB.
   4. Genomic analyses will be carried out at UoB
   5. MRI Imaging will be performed in the inpatient MRI department, UHB.
   6. Trans-cranial Magnetic Stimulation, EEG and TOMM will be conducted in the outpatients neurophysiology department of UHB.

Table 1. Schedule of sample analysis

| Time | Sample | Measurement |
| --- | --- | --- |
| 0 (point of injury) | 20mls venous blood | Biomarkers analysis |
| Day 1 (UHB ED) (if no point of injury sample taken) | 20mls venous blood | Biomarkers analysis |
| Day 1 (6-12hrs post injury) | 20mls venous blood | Biomarkers analysis |
| Day 3 | 20mls venous blood | Biomarkers analysis |
| Day 1-14 | Micro-diasylate  CSF (if available) | Biomarkers analysis |
| Day 180 (+/-30) | 20mls venous blood | Biomarkers analysis, Genetic analysis |

Table 2. Schedule of Imaging

| Time | Imaging | Notes |
| --- | --- | --- |
| Day 1-30 | MRI or MRS or DTI | Random selection of patients once stable |
| Day 180 (+/-30) | MRI or MRS or DTI | Same modality as before for comparisom |
| Day 360 (+/-30) | MRS | If 180 day MRS scan abnormal |

Table 3. Schedule of Outcome Measures

| Time | Measures | Notes |
| --- | --- | --- |
| Day 1-30 | Pupillometry | Where possible |
| Day 180 (+/-30) | Cognitive/psychological questionnaires |  |
| Day 360 (+/-30) | Cognitive/psychological questionnaires  TMS  ERP |  |

**Statistical Analysis**

Data will be analysed using SPSS. Descriptive statistics will be determined on parametric and non-parametric data. The independent variables are age, sex, ISS/NISS, type of injury, MTOR activity status. The primary dependent variables include biomarker levels, imaging analysis, TMS threshold and cognitive/psychological scoring. Exploratory assessment of data will be completed using analysis of co-variance and ordinal analysis of outcome. The spread of the means will be given as standard error of the mean. Differences in the means are considered significant if p<0.05. Psychological and cognitive scoring will undergo correlation analysis using Spearman’s rank correlation coefficient. They will also be subject to Rasch analysis. Multivariate regression analysis will be used to identify factors with significant impact on outcome.

**Outcome Measures**

Outcome of brain trauma will be assessed with cognitive and psychological symptom scoring, TMS threshold measurement and ERP. Initial severity scoring will be assessed with ISS/NISS. Changes in biomarkers will be measured in relation to brain trauma versus non-brain trauma and related to the above outcome assessment.

The primary outcome will be functional status as measured by the Glasgow Outcome Score at 6 months. Secondary outcome measures are functional status as assessed by the Glasgow Outcome Score at 12 months; cognitive and psychological symptom scoring with Post­concussion Symptom Questionnaire­19 (PCSQ­19), Impact of Events Scale – revised (IES­r), Depression and Anxiety Symptom Scale – 21 (DASS­21), PTSD checklist (PCL), Patient Health Questionnaire (PHQ), neurological assessment battery NAB-SM.

Other seconday outcome measures are neurophysiology measures of TMS threshold and Event related potentials.

Developing a better understanding of the relationships surrounding severe brain trauma and biomarkers in development will lead to better clinical understanding, categorisation, prognostication and treatments, both in targeting existing interventions and through development of new treatment in future interventional studies.

**Ethical Considerations**

In the initial phases of management it will be very challenging to obtain informed consent due to the nature of the injuries both in brain and non-brain trauma. Inherent in the severity of these injuries patients will frequently lack capacity under the Mental Capacity Act (MCA)2005. In the emergency setting a sample will be withdrawn from patients unable to consent under the MCA ‘waiver’ for research in emergency situations, subject to a favourable REC opinion. As soon as practicable and once a patient has been assessed under section 3 of the MCA the study team will seek the advice of a consultee prior to entry into the study (this will be under the terms of Section 32 and 33 of MCA 2005). Once capacity has been regained consent will be sought from the patient prior to continuation in the study.

The risks of participation in the study are minimal. The burden will be greatest physically and psychologically late in the study during rehabilitation.

Blood sampling is a routine aspect of hospital care. The drawing of samples will where possible be taken additionally to routine sampling of clinical necessity. At point of injury sampling will only take place where venous access has been placed for clinical reasons. Later in the study sampling will be performed where there is no clinical need. This will present minor discomfort to the participant. All sampling will be conducted by medical personnel well trained in the procedure with care taken to minimise discomfort.

Micro-dialysis is a routine aspect of care for head injuries at UHB. Currently the diasylate has no clinical use and is disposed of. Where micro-dialysis has been utilised for routine clinical treatment we propose to recover the diasylate for research analysis. CSF studies are occasionally performed for clinical reasons. Where these take place we propose to withdraw and additional 5mls for research purposes. Neither of these samplings would introduce an additional burden.

Imaging is a routine part of trauma care however at present CT scanning is the standard at initial assessment and following clinical changes that so warrant it. During the initial 1-4weeks of the hospital stay and following discharge from ITU participants will be randomised to an imaging modality and will undergo an additional scan. This will either be MRI using conventional sequences, MRS or DTI. Requirements for scans would not be in excess of the burden of normal MRI clinical scanning, there is no ionising radiation exposure and no known health concerns from exposure to magnetic fields. Exclusion criteria for exposure to magnetic fields will be applied as for clinical scanning. Scans would be repeated at 6 months using the same modality, and additionally at 1 year where 6 month MRS scans are abnormal.

Trans-cranial Magnetic stimulation is a neurophysiological technique that allows the induction of a small current in the brain to excite its neurons. It uses a magnetic field to pass through the scalp and the skull safely and painlessly. This technique, when conducted in accordance with internationally recognised guideline, is safe and is part of standard clinical tests in neurology in many countries worldwide. All participants will be screened for contraindications to TMS using a validated questionnaire for healthy participants that we have modified for traumatic brain injury (Annex k).

Electro-encephalogram and event related potential is a technique using conventional EEG recording in the setting of provision of controlled stimuli with the intent of measuring the p300 potential. The participant has an EEG recorded whilst undertaking a task with an audible odd-ball paradigm. This challenges the person with and unexpected ‘noise’ and the EEG records the cortical response. Changes in the timing of the well categorised p300 potential give us insight into the presence of damage in the sub-serve neural pathways.

Cognitive and psychological symptom scoring will be conducted in two parts. The first are a range of questionnaires used in routine practice. At follow-up a trained clinical psychologist will administer the remaining tests, TOMM and NAB, verbally.

Every effort will be made to take samples during procedures for clinical necessity, additional samples and testing will be timed to coincide when called for clinical follow-up. The testing will be accomplished in a single visit. There will however be occasions where patients would be lost to follow-up should they not be called in to allow testing and sampling. These instances will be minimised. Reasonable travel and parking costs will be reimbursed. Participants will be contacted via telephone, letter of email as per their preference following hospital discharge.

The study will be conducted in accordance with this protocol and Good Clinical Practice. The study complies, and will at all times comply, with the Declaration of Helsinki as adopted at the 52nd WMA General Assembly, Edinburgh, October 2000; and with the Draft Additional Protocol to the Council of Europe Convention on Human Rights and Biomedicine on Biomedical Research (CDBI/INF (2001) 5 dated 18 July 2001).

**Documentation**

All study documents appear at the end of this document.

Participant’s General Practitioners will be informed of participation in this study through the GP Letter, to include the Participants’ Information Leaflet.

**Publication**

Results will be published in peer-review journals and presented at relevant scientific meetings. All participants shall receive a short report of the study findings.

**Annexes**

1. Instructions for research team.
2. Guidelines for blood sampling.
3. Participant Information sheet - TBI. Dated 27 December 2013.
4. Participant Information sheet - continue in study. Dated 27 December 2013.
5. Participant Information sheet - Control. Dated 27 December 2013.
6. Consultee (Next of Kin) Information Sheet. Dated 27 December 2013.
7. Consent form for participants. Dated 27 December 2013.
8. Consent form for participants – continue in study following consultee advice. Dated 27 December 2013.
9. Record of Consultation for Consultee (Next of kin) of Participants Dated 27 December 2013.
10. GP information letter. Dated 27 December 2013.
11. Trans-cranial Magnetic Stimulation safety questionnaire.
12. Psychologist administered TOMM score sheet.
13. Participant Information Sheet - Supplement. Dated 27 December 2013.
