# Supplementary material for: Prehospital immune responses and development of multiple organ dysfunction syndrome following traumatic injury: A prospective cohort study
Source: PLoS Med. 2017 Jul 18;14(7):e1002338. doi: 10.1371/journal.pmed.1002338 (PMC5515405; doi:10.1371/journal.pmed.1002338)
Supplement: S2 Text — (DOC) [file pmed.1002338.s006.doc]

STROBE Statement—Checklist of items that should be included in reports of ***cohort studies***

|  | Item No | Recommendation | Page |  |  |
| --- | --- | --- | --- | --- | --- |
| **Title and abstract** | 1 | (*a*) Indicate the study’s design with a commonly used term in the title or the abstract | Title |  |  |
|  |  | (*b*) Provide in the abstract an informative and balanced summary of what was done and what was found | Abstract |  |  |
| Introduction | | |  |  |  |
| Background/rationale | 2 | Explain the scientific background and rationale for the investigation being reported | Introduction, Paragraphs 1-3 |  |  |
| Objectives | 3 | State specific objectives, including any prespecified hypotheses | Introduction,  Paragraph 4 |  |  |
| Methods | | |  |  |  |
| Study design | 4 | Present key elements of study design early in the paper | Methods  Paragraph 1 |  |  |
| Setting | 5 | Describe the setting, locations, and relevant dates, including periods of recruitment, exposure, follow-up, and data collection | Methods  Paragraphs 2-4, 6 |  |  |
| Participants | 6 | (*a*) Give the eligibility criteria, and the sources and methods of selection of participants. Describe methods of follow-up | Methods  Paragraphs 2-4, 6 |  |  |
|  |  | (*b*) For matched studies, give matching criteria and number of exposed and unexposed |  |  |  |
| Variables | 7 | Clearly define all outcomes, exposures, predictors, potential confounders, and effect modifiers. Give diagnostic criteria, if applicable | Methods  Paragraph 15 |  |  |
| Data sources/ measurement | 8* | For each variable of interest, give sources of data and details of methods of assessment (measurement). Describe comparability of assessment methods if there is more than one group | Methods  Paragraphs 1-16 |  |  |
| Bias | 9 | Describe any efforts to address potential sources of bias | Methods  Paragraph 2 |  |  |
| Study size | 10 | Explain how the study size was arrived at | Methods  Paragraph 16 |  |  |
| Quantitative variables | 11 | Explain how quantitative variables were handled in the analyses. If applicable, describe which groupings were chosen and why | Methods  Paragraph 16 |  |  |
| Statistical methods | 12 | (*a*) Describe all statistical methods, including those used to control for confounding | Methods  Paragraph 16 |  |  |
|  |  | (*b*) Describe any methods used to examine subgroups and interactions | Methods  Paragraph 16 |  |  |
|  |  | (*c*) Explain how missing data were addressed | Methods  Paragraph 16 |  |  |
|  |  | (*d*) If applicable, explain how loss to follow-up was addressed |  |  |  |
|  |  | (*e*) Describe any sensitivity analyses | Methods  Paragraph 16 |  |  |
| Results | | |  |  |  |
| Participants | 13* | (a) Report numbers of individuals at each stage of study—eg numbers potentially eligible, examined for eligibility, confirmed eligible, included in the study, completing follow-up, and analysed | Results  Fig 1, S2 Fig. |  |  |
|  |  | (b) Give reasons for non-participation at each stage | Results  Fig 1, S2 Fig. |  |  |
|  |  | (c) Consider use of a flow diagram | Results  Fig 1, S2 Fig. |  |  |
| Descriptive data | 14* | (a) Give characteristics of study participants (eg demographic, clinical, social) and information on exposures and potential confounders | Results  Paragraph 1,  Table 1 |  |  |
|  |  | (b) Indicate number of participants with missing data for each variable of interest | Results  Fig 1, S2 Fig. |  |  |
|  |  | (c) Summarise follow-up time (eg, average and total amount) | Results  Fig 1, S2 Fig. |  |  |
| Outcome data | 15* | Report numbers of outcome events or summary measures over time | Methods  Paragraph 15;  Results  S2 Fig. |  |  |
| Main results | 16 | (*a*) Give unadjusted estimates and, if applicable, confounder-adjusted estimates and their precision (eg, 95% confidence interval). Make clear which confounders were adjusted for and why they were included | Results  Paragraph 17,18  Table 9, 10 |  |  |
|  |  | (*b*) Report category boundaries when continuous variables were categorized | Results  Table 1 |  |  |
|  |  | (*c*) If relevant, consider translating estimates of relative risk into absolute risk for a meaningful time period |  |  |  |
| Other analyses | 17 | Report other analyses done—eg analyses of subgroups and interactions, and sensitivity analyses |  |  |  |
| Discussion | | |  |  |  |
| Key results | 18 | Summarise key results with reference to study objectives | Discussion  Paragraph 1 |  |  |
| Limitations | 19 | Discuss limitations of the study, taking into account sources of potential bias or imprecision. Discuss both direction and magnitude of any potential bias | Discussion  Paragraph 9 |  |  |
| Interpretation | 20 | Give a cautious overall interpretation of results considering objectives, limitations, multiplicity of analyses, results from similar studies, and other relevant evidence | Discussion  Paragraphs 1-10 |  |  |
| Generalisability | 21 | Discuss the generalisability (external validity) of the study results | Discussion  Paragraphs 7,9 |  |  |
| Other information | | |  |  |  |
| Funding | 22 | Give the source of funding and the role of the funders for the present study and, if applicable, for the original study on which the present article is based | Financial disclosure statement |  |  |

*Give information separately for exposed and unexposed groups.

**Note:** An Explanation and Elaboration article discusses each checklist item and gives methodological background and published examples of transparent reporting. The STROBE checklist is best used in conjunction with this article (freely available on the Web sites of PLoS Medicine at http://www.plosmedicine.org/, Annals of Internal Medicine at http://www.annals.org/, and Epidemiology at http://www.epidem.com/). Information on the STROBE Initiative is available at http://www.strobe-statement.org.
